# Tau reduction with artificial microRNAs modulates neuronal physiology and improves tauopathy phenotypes in mice

**DOI:** 10.1016/j.ymthe.2024.01.033

**Published:** 2024-02-03

**Authors:** Carolina Lucía Facal, Iván Fernández Bessone, Javier Andrés Muñiz, A. Ezequiel Pereyra, Olivia Pedroncini, Indiana Páez-Paz, Ramiro Clerici-Delville, Cayetana Arnaiz, Leandro Urrutia, Germán Falasco, Carla Verónica Argañaraz, Trinidad Saez, Antonia Marin-Burgin, Mariano Soiza-Reilly, Tomás Falzone, María Elena Avale

**Affiliations:** 1 Instituto de Investigaciones en Ingeniería Genética y Biología Molecular (INGEBI), CONICET, Buenos Aires, Argentina; 2 Instituto de Biología Celular y Neurociencias (IBCN), Universidad de Buenos Aires, CONICET-UBA, Buenos Aires, Argentina; 3 Instituto de Investigación en Biomedicina de Buenos Aires (IBioBA), Partner Institute of the Max Planck Society, CONICET-MPSP, Buenos Aires, Argentina; 4 Centro de imágenes Moleculares, FLENI, Buenos Aires, Argentina; 5 Instituto de Fisiología Biología Molecular y Neurociencias (IFIBYNE), Universidad de Buenos Aires, CONICET-UBA, Buenos Aires, Argentina

**Keywords:** tau, tauopathy, neurodegeneration, microRNAs, RNA therapy, htau mice, MAPT knockdown, gene therapy

## Abstract

Abnormal tau accumulation is the hallmark of several neurodegenerative diseases, named tauopathies. Strategies aimed at reducing tau in the brain are promising therapeutic interventions, yet more precise therapies would require targeting specific nuclei and neuronal subpopulations affected by disease while avoiding global reduction of physiological tau. Here, we developed artificial microRNAs directed against the human *MAPT* mRNA to dwindle tau protein by engaging the endogenous RNA interference pathway. In human differentiated neurons in culture, microRNA-mediated tau reduction diminished neuronal firing without affecting neuronal morphology or impairing axonal transport. In the htau mouse model of tauopathy, we locally expressed artificial microRNAs in the prefrontal cortex (PFC), an area particularly vulnerable to initiating tau pathology in this model. Tau knockdown prevented the accumulation of insoluble and hyperphosphorylated tau, modulated firing activity of putative pyramidal neurons, and improved glucose uptake in the PFC. Moreover, such tau reduction prevented cognitive decline in aged htau mice. Our results suggest target engagement of designed tau-microRNAs to effectively reduce tau pathology, providing a proof of concept for a potential therapeutic approach based on local tau knockdown to rescue tauopathy-related phenotypes.

## Graphical abstract

**Figure:**
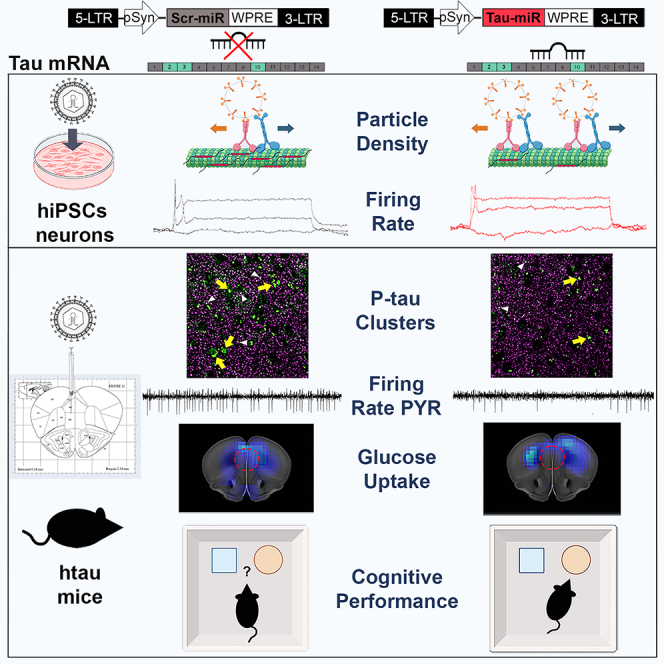

## Teaser

Abnormal accumulation of tau protein is the hallmark of neurodegenerative diseases called tauopathies. Reducing tau burden in the brain is a plausible therapeutic approach. In this study, Avale and colleagues developed artificial microRNAs that block tau synthesis and showed that they prevent pathological phenotypes in a mouse model of tauopathy.

## Introduction

Tauopathies encompass several neurodegenerative diseases related to abnormal tau protein metabolism, including Alzheimer’s disease (AD), frontotemporal lobar degeneration, progressive supranuclear palsy, and corticobasal degeneration.^1^ Each tauopathy has its unique clinical features, affecting specific vulnerable neurons within brain nuclei and showing defined propagation patterns.^2^^,^^3^^,^^4^

Tau is a microtubule-associated protein, involved in microtubule dynamics and axonal transport.^5^^,^^6^^,^^7^ In tauopathies, tau mislocalizes from the axon to the somatodendritic compartment where it accumulates in insoluble neurofibrillary tangles (NFTs).^8^^,^^9^ This process might arise due to a myriad of gene mutations, abnormal post-transcriptional processing, pathological hyperphosphorylation, and/or clearance deficits.^10^^,^^11^ Tau physiology is highly regulated by dynamic phosphorylation and dephosphorylation processes.^12^ In AD and other tauopathies, tau becomes hyperphosphorylated at specific pathological epitopes,^7^^,^^11^^,^^13^^,^^14^ which triggers its dissociation from microtubules and its mislocalization to the neuronal soma.^15^^,^^16^^,^^17^ These pathological events lead to neuronal impairments that could begin years before neuronal death.^18^ Each tauopathy correlates with specific abnormal tau species^19^ that originate in defined brain nuclei.^20^^,^^21^ Such aberrant (pathological) forms of tau include hyperphosphorylated, misfolded, or truncated tau, as well as oligomers and seeding-competent tau, which can spread from affected neurons to other brain areas.^2^^,^^4^^,^^22^ Therefore, targeting the accumulation of tau in early affected nuclei could be an efficient strategy to prevent disease progression.^2^^,^^4^^,^^23^^,^^24^

The most plausible therapeutic approaches tested so far for tauopathies point to reduce tau burden.^11^^,^^24^^,^^25^^,^^26^^,^^27^^,^^28^ Among them stand out antibody-based immunotherapies that facilitate tau clearance^23^^,^^29^ and antisense oligonucleotides (ASOs) directed to tau mRNA, which demonstrated effectiveness in reducing tau pathology in preclinical studies.^30^ Both therapies are currently under clinical trials. Although these strategies are very promising, the main bottleneck is that they require successive administrations, besides the possible side effects due to global tau reduction throughout the brain. In this sense, recent evidence showing selective vulnerability of specific neurons and brain nuclei to initiate tau pathology^20^^,^^31^ opens up a new therapeutic window to develop tailored approaches, which can specifically reduce tau into affected brain structures.

As a proof of concept, we aimed to locally reduce pathological tau accumulation in the prefrontal cortex (PFC) of a mouse model of tauopathy at early stages of disease. To this end, we developed artificial microRNAs (miRNAs) that target the human *MAPT* transcript and lower tau protein synthesis. miRNAs play a crucial role in post-transcriptional gene regulation in the brain and other tissues. Natural miRNAs can be modified to target a specific mRNA and reduce the synthesis of a given protein via the endogenous RNA interference pathway.^32^ The expression of the siRNA target sequence within a natural scaffold miRNA leads to an efficient production of silencing molecules.^33^ Such laboratory-engineered artificial miRNAs can be stably expressed using viral vectors for long-term gene silencing *in vivo*.^32^ Moreover, the use of viral vectors to deliver artificial miRNAs restricts their expression only to virally transduced areas.

In this study, two artificial anti-tau-miRNAs showing target engagement *in vitro* were delivered by lentiviral vectors (LVs) into the PFC of a mouse model of tauopathy. Using behavioral tests, molecular and imaging studies, and electrophysiological recordings, we analyzed phenotypes of tau-related neurodegeneration to determine the outcome of local tau downregulation *in vivo*. In addition, we analyzed single-cell physiological effects of tau reduction in human neurons in culture. Our findings suggest that local, long-term expression of Tau-miRNAs prevents tau pathology and cognitive decline, and that modulation of electrophysiological activity might be critically related to the therapeutic benefits of tau reduction.

## Results

### Tau-miRNA validation in human neurons in culture

**Figure 1:**
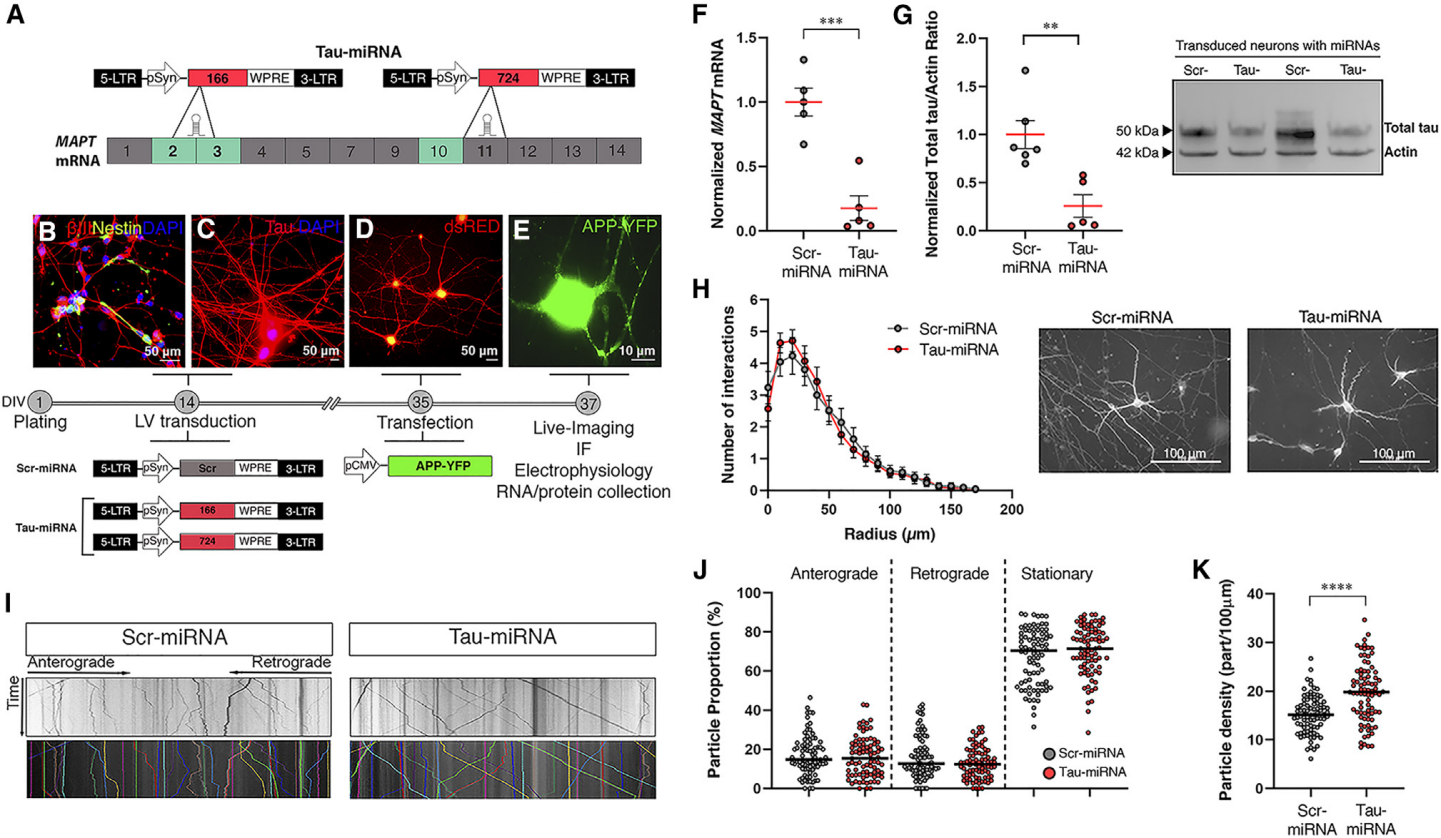
Tau-miRNA validation in human neurons in culture (A) Two artificial miRNAs were designed to target the *MAPT* mRNA. Tau-miRNA 166 miRNA is directed to the junction between exons 2/3. Tau-miRNA 724 targeted the constitutive exon 11. See also Figure S1. (B–E) Experimental design to analyze the effect of Tau-miRNA treatment in human derived neurons. Polarized human neuronal cultures at DIV14 immunostained for βIII-tubulin and Nestin (B, red and green, respectively) and Tau (C, red). Nuclei were counterstained with DAPI (blue). See also Figure S1. Neurons were transduced with LVs containing Scr-miRNA or an equimolar combination of Tau-miRNAs (166 + 724) and cotransduced with an LV carrying the dsRed fluorescent protein. After 3 weeks (DIV35), LV transduction was confirmed by dsRed expression (D), and neurons were transfected with a plasmid expressing the APP-YFP fusion protein to perform live imaging at DIV37 (E). (F) *MAPT* mRNA levels obtained by qPCR from transduced neurons with miRNAs. *GAPDH* mRNA was used as housekeeping for normalization. Scr-miRNA and Tau-miRNA n = 3 wells from two independent experiments; ∗∗∗p < 0.001, unpaired t test. Data are shown as scatter dot plots, with mean ± SEM. (G) Western blot detecting total tau protein content in Scr- or Tau-miRNA-transduced neurons. Left: quantification of tau optical density normalized to actin as loading control. Scr-miRNA and Tau-miRNA n = 3 wells from two independent experiments; ∗∗p < 0.01, unpaired t test. Data are shown as scatter dot plots, with mean ± SEM. Right: representative blot. See also Figure S1. (H) Left: quantification of projection intersections versus radius (in micrometers) obtained from Sholl analysis in miRNA-transduced neurons. Scr-miRNA n = 21, Tau-miRNA n = 28 neurons, from four independent experiments; not significant differences, two-sample Kolmogorov-Smirnov test. Data are shown as mean ± SEM. Right: representative images of neuronal arborization. (I) Representative kymographs obtained from 30-s videos (8 frames/s) recorded in axons from miRNA-transduced neurons transfected with APP-YFP. Colored lines indicate trajectories recovered by tracking system. (J and K) (J) Average proportion of anterograde, retrograde, and stationary APP vesicles and (K) total particle density, calculated per 100 μm of axonal length. Scr-miRNA n = 82, Tau-miRNA n = 83, from three independent experiments; ∗∗∗∗p < 0.0001; (J) Mann-Whitney U test and (K) unpaired t test. Data are shown as scatter dot plots, with median. See also Videos S1 and S2.

Two artificial miRNAs targeting the human *MAPT* transcript (Tau-miRNAs 166 and 724; Figure 1A) and a scrambled control (Scr-miRNA) were obtained *in silico* following an in-house design protocol and further curation to avoid off-target effects (see section “materials and methods”). Each Tau-miRNA, when used separately, showed efficient tau reduction in SH-SY5Y cells (Figure S1A); however, a more robust effect was observed when both were used in combination (1:1; Figure S1A). In addition, Tau-miRNA 166 targets the junction between exons 2 and 3, both of which can be alternatively spliced, while Tau-miRNA 724 targets constitutive exon 11 (Figure 1A). Therefore, we used both miRNAs to ensure reduction of all six tau isoforms produced in the adult brain from the *MAPT* gene. In all subsequent experiments Tau-miRNA 166 and Tau-miRNA 724 were used in equimolar combination (1:1) and are referred to as Tau-miRNA for simplification (see section “materials and methods”). All miRNAs used in this study were delivered by LVs under the synapsin promoter to restrict their expression to neurons.

We first determined the functional impact of reducing tau protein in human neurons in culture, differentiated from human induced pluripotent stem cells (hiPSCs).^34^^,^^35^ LV transduction with Tau-miRNA or Scr-miRNA was performed at day *in vitro* 14 (DIV14), a stage when neurons were polarized and exhibited elevated levels of neuronal markers such as BetaIII-tubulin and tau (Figures 1B, 1C, and S1B). Transduction efficiency was confirmed with dsRed fluorescent protein (LV-dsRED; Figure 1D). Considering kinetics of tau protein turnover in human neurons,^36^ all experiments were conducted 21–23 days after LV transduction, including transfection at DIV35 with a fluorescent amyloid precursor protein (APP-YFP; Figure 1E) to perform live-imaging studies prior to RNA and protein collection. Tau-miRNA transduction resulted in a significant ∼75% reduction of tau mRNA (Figure 1F) and protein levels (Figures 1G and S1B).

Since tau plays a role in mediating stability and dynamics of neuronal cytoskeleton, we assessed the impact of tau reduction on neuronal polarization. We performed a Scholl analysis to determine the number of neurite processes and arborization at various distances from the soma (see section “materials and methods”). Scholl analysis showed similar arborization between Tau-miRNA- and Scr-miRNA-treated human neurons (Figure 1H), indicating that no apparent morphological changes were induced by tau reduction. We next evaluated the effect of tau reduction on axonal transport. To this end, Tau-miRNA- or Scr-miRNA-transduced neurons were transfected at DIV35 with a fluorescent amyloid precursor protein (APP-YFP; Figure 1E) to perform live-imaging analysis (Figures 1I–1K; Videos S1 and S2). APP-YFP vesicle transport data were extracted to track vesicle dynamics^37^ (see section “materials and methods”). Tau reduction alteredneither the proportion of vesicles moving in the anterograde or retrograde direction nor the proportion of stationary vesicles (Figure 1J). However, an increase in vesicle density within axons was observed in Tau-miRNA-transduced neurons (Figure 1K), suggesting that tau decrease might favor the vesicle recruitment to the axon. Together, these results demonstrate the efficiency of Tau-miRNA to reduce endogenous tau expression in differentiated human neurons without dramatic effects on neuronal morphology or transport dynamics.

Video S1. Axonal transport in Scr-miRNA treated human differentiated neurons Representative live-imaging videos used for analyzing axonal transport dynamics in Scr-miRNA (Video S1) and Tau-miRNA (Video S2) groups, related to Figure 1.

Video S2. Axonal transport in Tau-miRNA treated human differentiated neurons Representative live-imaging videos used for analyzing axonal transport dynamics in Scr-miRNA (Video S1) and Tau-miRNA (Video S2) groups, related to Figure 1.

### Modulation of electrical properties of neurons upon tau knockdown

**Figure 2:**
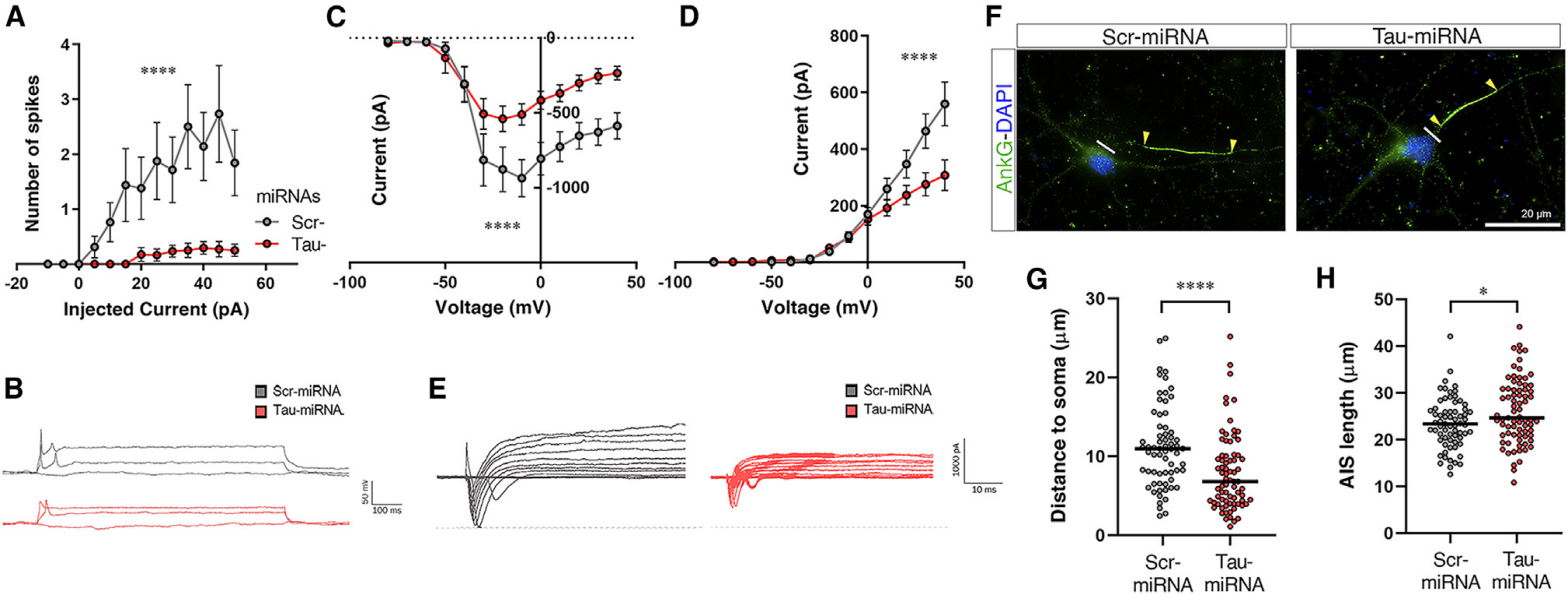
Modulation of electrical properties of neurons upon tau knockdown (A) Current-clamp configuration showing the average number of action potentials evoked by increasing steps of depolarizing current in miRNAs treated neurons. (B) Representative spiking traces. (C and D) Voltage-clamp configuration showing the average sodium (C) and potassium (D) current amplitudes (in pico-amperes) for transduced neurons with miRNAs. (E) Representative current traces obtained after increasing voltage steps. Scr-miRNA n = 21, Tau-miRNA n = 17, from three independent experiments; (A, C, and D) ∗∗∗∗p < 0.0001, two-way ANOVA, miRNAs factor. Data are shown as mean ± SEM. See also Figure S1. (F) Immunofluorescence images showing the AIS stained with AnkyrinG (AnkG, green) in miRNA-transduced neurons. (G and H) Quantitative analyses of distance to soma (G, line to first arrow in F) and length (H, arrow to arrow in F) of the AIS for both groups. Scr-miRNA n = 67, Tau-miRNA n = 75, from three independent experiments; ∗p < 0.05, ∗∗∗∗p < 0.0001, (G) Mann-Whitney U test and (H) unpaired t test. Data are shown as scatter dot plots, with median.

Based on growing evidence indicating the role of tau protein on neuronal firing *in vivo*,^38^^,^^39^ we aimed to test whether tau knockdown impairs neural cell intrinsic electrical properties in human neurons. We performed electrophysiological recordings after miRNA expression (Figures 2A–2E and S1D‒S1G). Patch-clamp recordings showed decreased number of spikes in Tau-miRNA neurons in response to applied current steps (Figures 2A and 2B), with diminished sodium and potassium currents (Figures 2C–2E). Moreover, reduced action potential amplitudes were detected after tau reduction (Figure S1E) while input resistance and threshold values were unaffected (Figures S1F and S1G). Since the position and plasticity of the axon initial segment (AIS) correlate with neuronal excitability,^40^ we investigated AIS dynamics in Scr-miRNA- and Tau-miRNA-treated human neurons using ankyrin-G staining (Figure 2F). Tau knockdown induced the relocation of the AIS closer to the soma (Figure 2G) and increased the AIS length (Figure 2H), suggesting a homeostatic mechanism to compensate the reduction of intrinsic neuronal firing in Tau-miRNA-transduced neurons. Together, these findings indicate that lowering tau protein modulates electrical properties of differentiated human neurons in culture.

### Tau-miRNA expression reduces tau pathology in the medial PFC of htau mice

We next aimed to determine if tau reduction in vulnerable nuclei of the adult brain could prevent tauopathy-related phenotypes. We used a well-stablished model of tauopathy, the htau mouse.^41^ hTau mice carry a full-length human *MAPT* transgene with H1 haplotype (which predisposes to tauopathies) in a murine tau knockout background. In these mice, the six isoforms of human tau are expressed in the adult brain at a 2.5-fold higher level compared to wild-type (WT) mice.^41^ The htau model recapitulates key tauopathy phenotypes, including age-associated tau hyperphosphorylation and pathological tau accumulation in the prefrontal cortex (PFC), which correlates with cognitive deficits^42^^,^^43^^,^^44^ and motor coordination deficits.^45^ In addition, these mice present age-related changes in glucose uptake throughout the brain revealed by PET scans, showing that the PFC is severely affected.^44^

**Figure 3:**
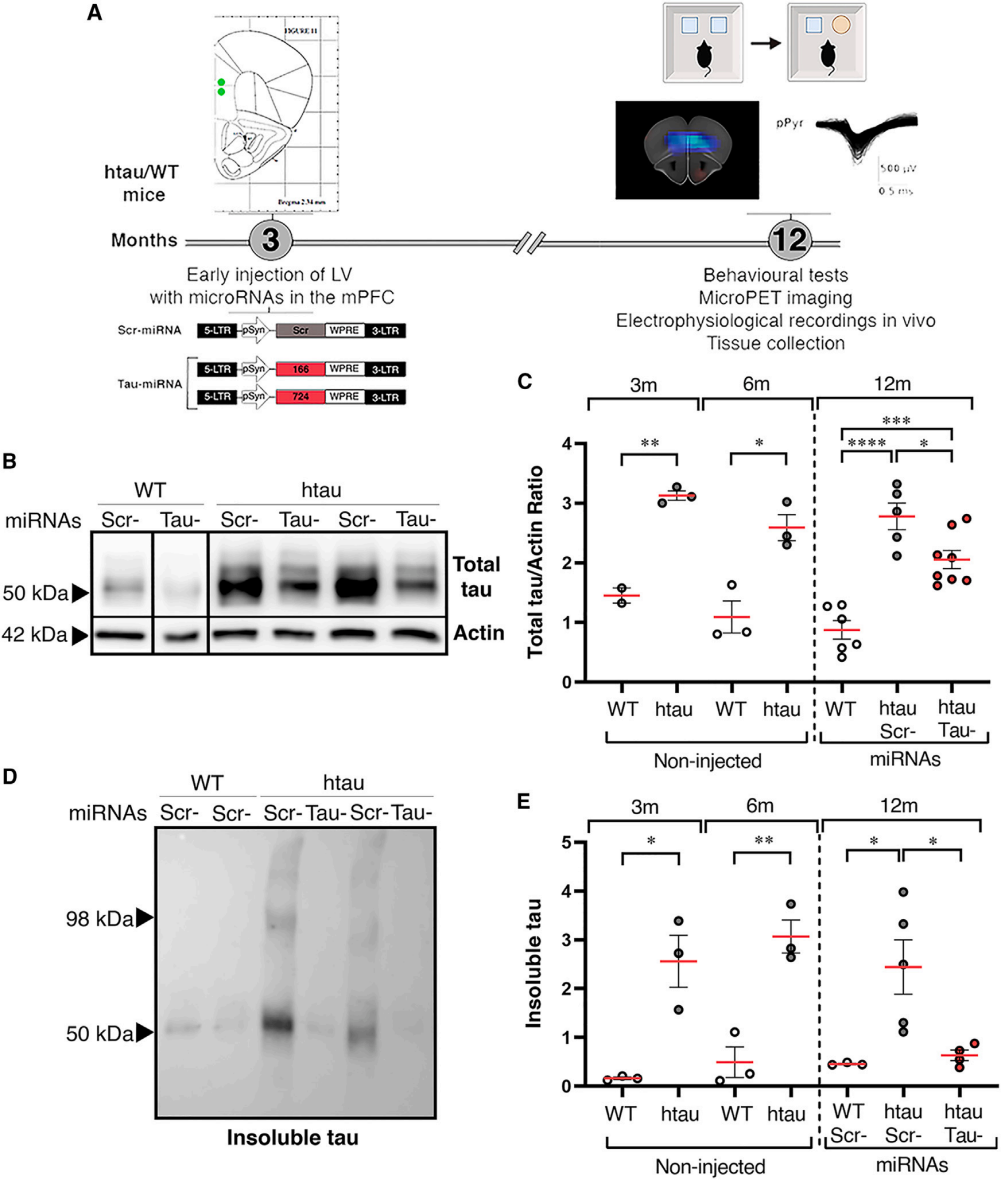
Tau-miRNA reduces total and insoluble tau in the mPFC of htau mice (A) Workflow of lentiviral transduction with miRNAs in the mPFC of mice. Three-month-old htau and WT littermates were injected into the prelimbic area of the mPFC with LVs containing Scr-miRNA or an equimolar combination of Tau-miRNAs (166 + 724). At 12 months old, behavioral analyses, 18F-FDG-PET imaging, and electrophysiological recordings were performed. Subsequently, tissue was collected for biochemical analyses. (B) Representative immunoblots of total tau protein contents in the mPFC of miRNA-injected mice. (C) Quantification of total tau protein contents in non-injected mice (left) and miRNA-injected groups (right). Non-injected 3m: WT n = 2, htau n = 3; 6m: WT n = 3, htau n = 3. miRNAs injected 12m: WT Scr n = 6 and Tau n = 6, htau Scr n = 5, htau Tau-miRNA n = 8. ∗p < 0.05, ∗∗p < 0.01, ∗∗∗p < 0.001, ∗∗∗∗p < 0.0001; 3m and 6m unpaired t test and 12m one-way ANOVA followed by Tukey’s *post hoc* test. Data are shown as scatter dot plots, with mean ± SEM. See also Figure S2. (D) Representative immunoblot of insoluble tau contents in the mPFC of miRNA-injected mice. (E) Quantification of insoluble tau contents in the mPFC of non-injected mice (left) and miRNA-injected groups (right). Non-injected 3m: WT n = 3, htau n = 3; 6m WT n = 3, htau n = 3, miRNAs injected 12m: WT Scr-n = 2, htau Scr-n = 5, htau Tau-miRNA n = 4; ∗p < 0.05, ∗∗p < 0.01; 3m and 6m unpaired t test and 12m one-way ANOVA followed by Tukey’s *post hoc* test. Data are shown as scatter dot plots, with mean ± SEM. See also Figure S2.

For this study, adult htau mice were injected at 3 months old with Tau-miRNA or Scr-miRNA into the medial PFC (mPFC) and analyses were performed to evaluate tau pathology and related phenotypes in aged htau mice, at 12 months old (Figure 3A). As previously reported, htau mice showed increased tau contents in the PFC compared to WT littermates (Figures 3C and S2A), which correlates with insoluble tau accumulation. In htau mice injected with Tau-miRNA, a 30% of total tau reduction was observed in the mPFC at 12 months old (Figures 3B, 3C, and S2A). In addition, sarkosyl insolubility assay was conducted to evaluate the content of insoluble tau species in the mPFC of htau mice. A dramatic decrease of insoluble tau contents was detected in htau mice injected with Tau-miRNA in the mPFC (Figures 3D, 3E, and S2B), indicating that local targeting of *MAPT* mRNA can lead to an effective reduction of pathological tau accumulation. To confirm that Tau-miRNA silencing was specifically restricted to the area of injection, total tau contents were also evaluated in the adjacent motor cortex 1 (M1). No significant differences were observed between htau mice injected with Scr-miRNA or Tau-miRNA, confirming that tau reduction was locally directed to the mPFC (Figure S2C).

**Figure 4:**
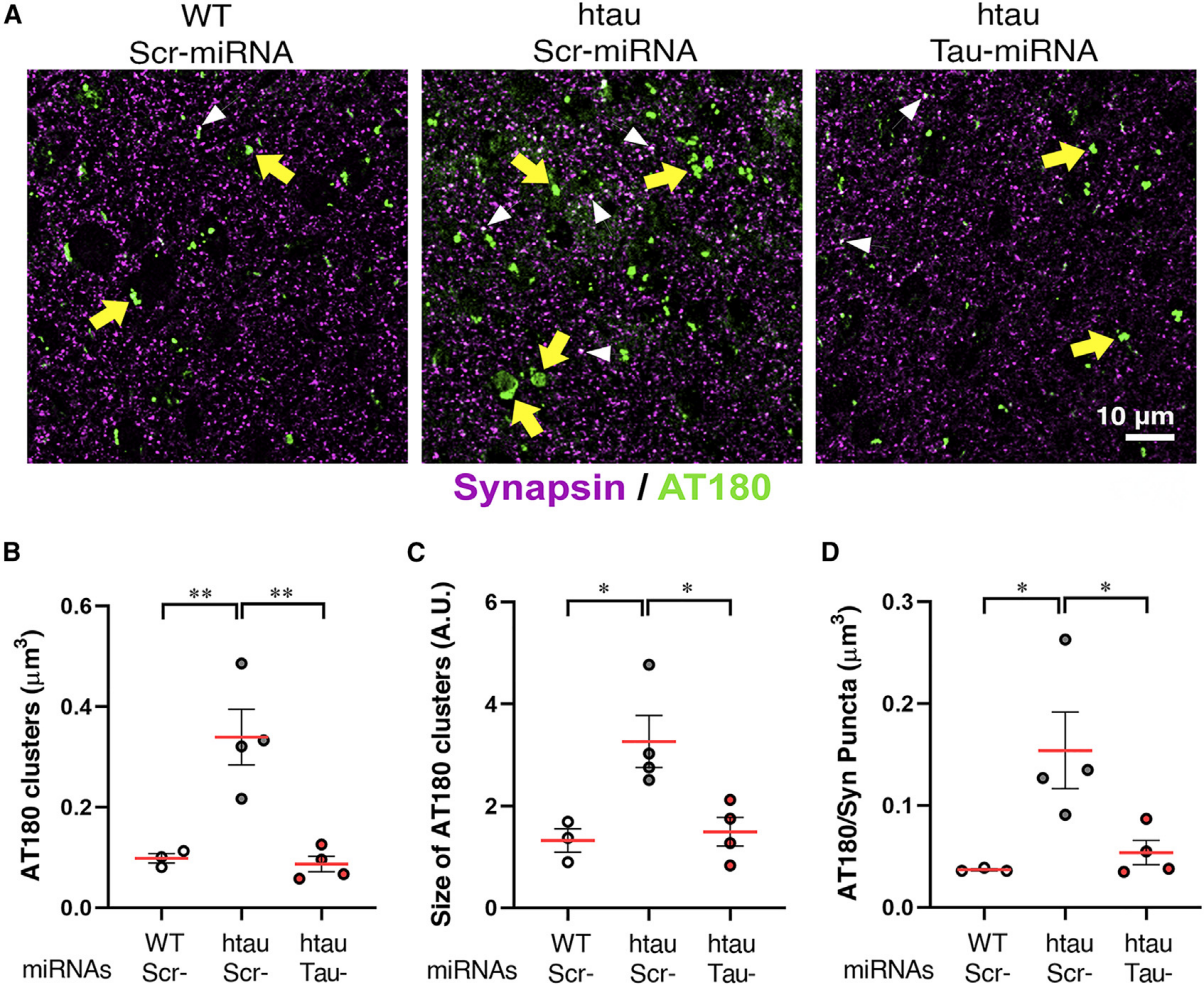
Tau-miRNA reduces p-tau clusters in the mPFC of htau mice (A) Representative images obtained by array tomography, showing phospho-tau (AT180, green) in the mPFC synaptic neuropil of miRNA-injected mice. Arrows indicate colocalization of AT180 with the presynaptic marker synapsin 1a (magenta). (B and C) Quantitative analyses of density (B) and relative size (C) of AT180 clusters in the mPFC. (D) Density of AT180/synapsin colocalized puncta in the mPFC. WT-Scr-n = 3, htau-Scr n = 4, htau-Tau n = 4; ∗p < 0.05, ∗∗p < 0.01, one-way ANOVA followed by Tukey’s *post hoc* test. Data are shown as scatter dot plots, with mean ± SEM. See also Figure S3.

To further investigate whether Tau-miRNA expression could prevent the accumulation of pathological phosphorylated tau, we analyzed phospho-tau (p-tau) clusters in prefrontal neurons of htau and WT mice using high-resolution immunofluorescence array tomography (Figure 4). Compared to WT, htau control mice showed increased number, density and size of p-Tau clusters (Figures 4A–4C) and of p-tau/synapsin colabeling in presynaptic terminals (Figures 4A and 4D), which were prevented in htau mice injected with Tau-miRNA (Figures 4A–4D). Interestingly, aged htau mice did not display changes in glutamatergic or in GABAergic synaptic boutons in the mPFC when compared to WT controls (Figures S3A and S3B). However, increases in VGlut1 and VGAT synaptic boutons were detected in the Tau-miRNA htau group (Figures S3A and S3B), suggesting the occurrence of possible synaptic changes related to tau knockdown. To determine whether this effect of Tau-miRNAs was restricted to the PFC, we also analyzed the agranular insular cortex (AI), an area that localizes near the site of injection. Tau-miRNA expression did not affect the density (Figure S3D), relative size (Figure S3E), or p-tau/synapsin colabeling puncta (Figure S3F) in the AI of htau mice, also confirming a localized mechanism of action by artificial tau-targeting miRNAs.

### Tau reduction modulates firing of pyramidal neurons in the mPFC of htau mice

**Figure 5:**
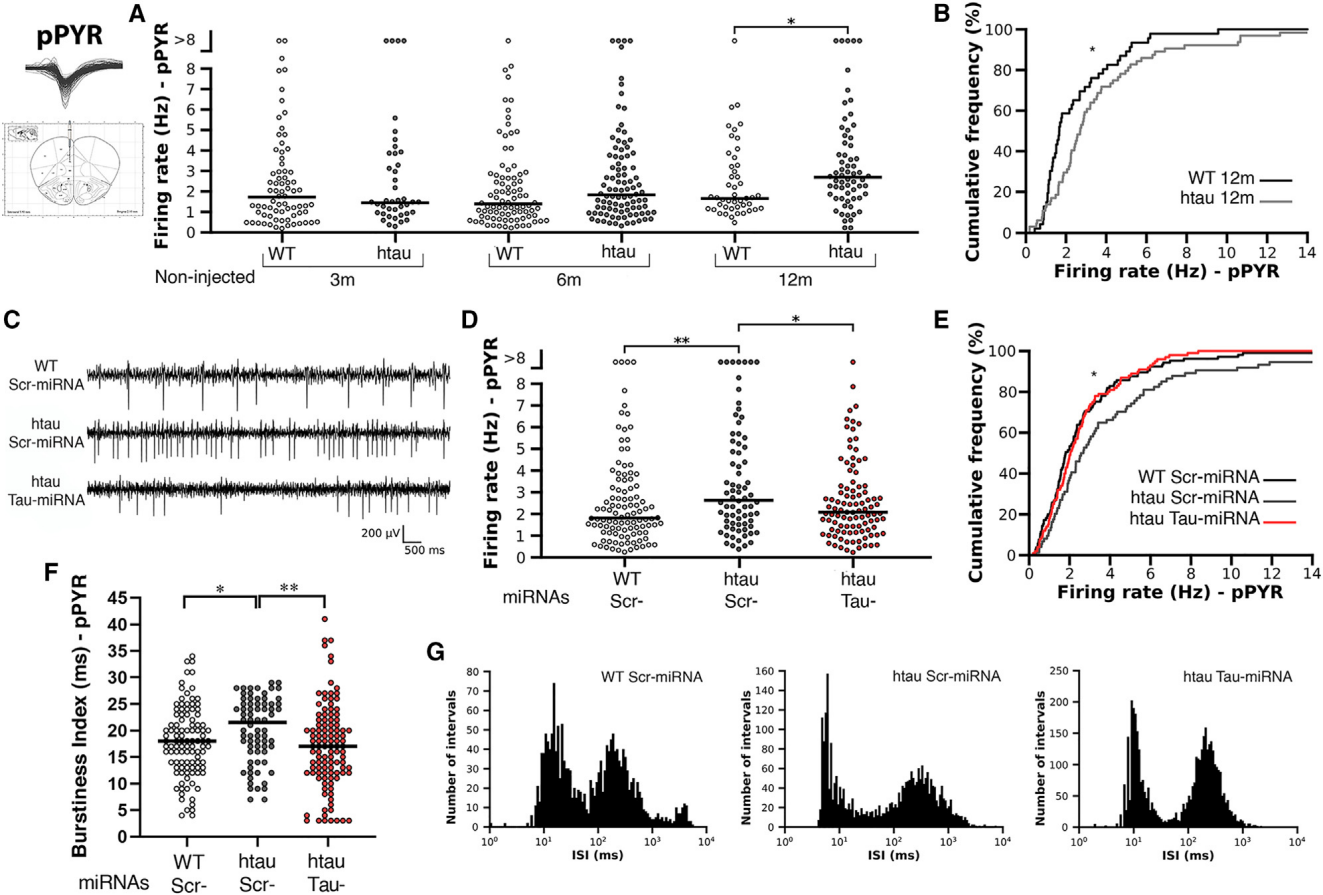
Tau-miRNA modulates firing of pyramidal neurons in the mPFC of htau mice (A) Temporal curse of firing rates from putative pyramidal neurons (pPYRs) in the mPFC of non-injected WT and htau mice at 3, 6, and 12 months of age. 3m, WT n = 73 neurons/four mice, htau n = 41 neurons/three mice; 6m, WT n = 87 neurons/four mice, htau n = 94 neurons/four mice; 12m, WT n = 46 neurons/three mice, htau n = 64 neurons/six mice; ∗p < 0.05, Mann-Whitney U test. Each dot represents the mean firing rate of each recorded neuron along the session. Black lines indicate the median value per group. (B) Cumulative frequency for firing rates of pPYR from non-injected, 12-month-old mice. ∗p < 0.05, two-sample Kolmogorov-Smirnov test. See also Figure S4. (C) Representative traces of an electrophysiological signal band-pass filtered between 300 and 6,000 Hz for miRNA-injected groups. (D) Firing rate of pPYRs in the mPFC of miRNA-injected mice. WT Scr n = 105 neurons/four mice, htau Scr n = 74 neurons/five mice, htau Tau n = 100 neurons/seven mice; ∗p < 0.05, ∗∗p < 0.01, Mann-Whitney U test. Each dot represents the mean firing rate of each recorded neuron along the session. Black lines indicate the median value per group. (E) Cumulative frequency for firing rates of pPYR from treated groups. ∗p < 0.05, two-sample Kolmogorov-Smirnov test. (F) Burstiness index of pPYR in the mPFC of miRNA-injected mice. ∗p < 0.05, ∗∗p < 0.01, Mann-Whitney U test. Each dot represents the mean firing rate of each recorded neuron along the session. Black lines indicate the median value per group. (G) ISI histograms plotted on a log scale for a single representative neuron from miRNA-injected groups. The clustering of spikes in two peaks indicates that action potential occurred in bursts in these selected neurons. See also Figure S4.

Based on previous reports showing that pathological tau accumulation affects neuronal excitability in different models of tauopathy,^44^^,^^45^^,^^46^^,^^47^ we first characterized the temporal course of neuronal firing in the prelimbic area of htau mice, discriminating between putative pyramidal neurons and interneurons based on their spike waveforms (see section “materials and methods” and Figure S4A). Indeed, compared to aged-matched WT siblings, htau mice showed a significant increase in firing rates of pyramidal neurons between 6 and 12 months old (Figures 5A and 5B). Interestingly, such increase was prevented in htau mice injected with Tau-miRNA (Figures 5C–5E), although no significant changes were observed in firing rates of mPFC interneurons either in htau mice during aging (Figures S4B‒S4C) or after Tau-miRNA injection (Figures S4D‒S4E). Moreover, burstiness index and interspike interval (ISI) of prelimbic pyramidal neurons presented similar distributions between htau injected with Tau-miRNA and WT mice (Figures 5F and 5G). Together, these data suggest that local tau downregulation in the mPFC of htau mice prevents changes in firing rates of pyramidal neurons, consistent with previous reports using other tau-reducing strategies in mice models.^48^

### Tau reduction in the mPFC prevents cognitive decline and deficits in brain metabolism in htau mice

**Figure 6:**
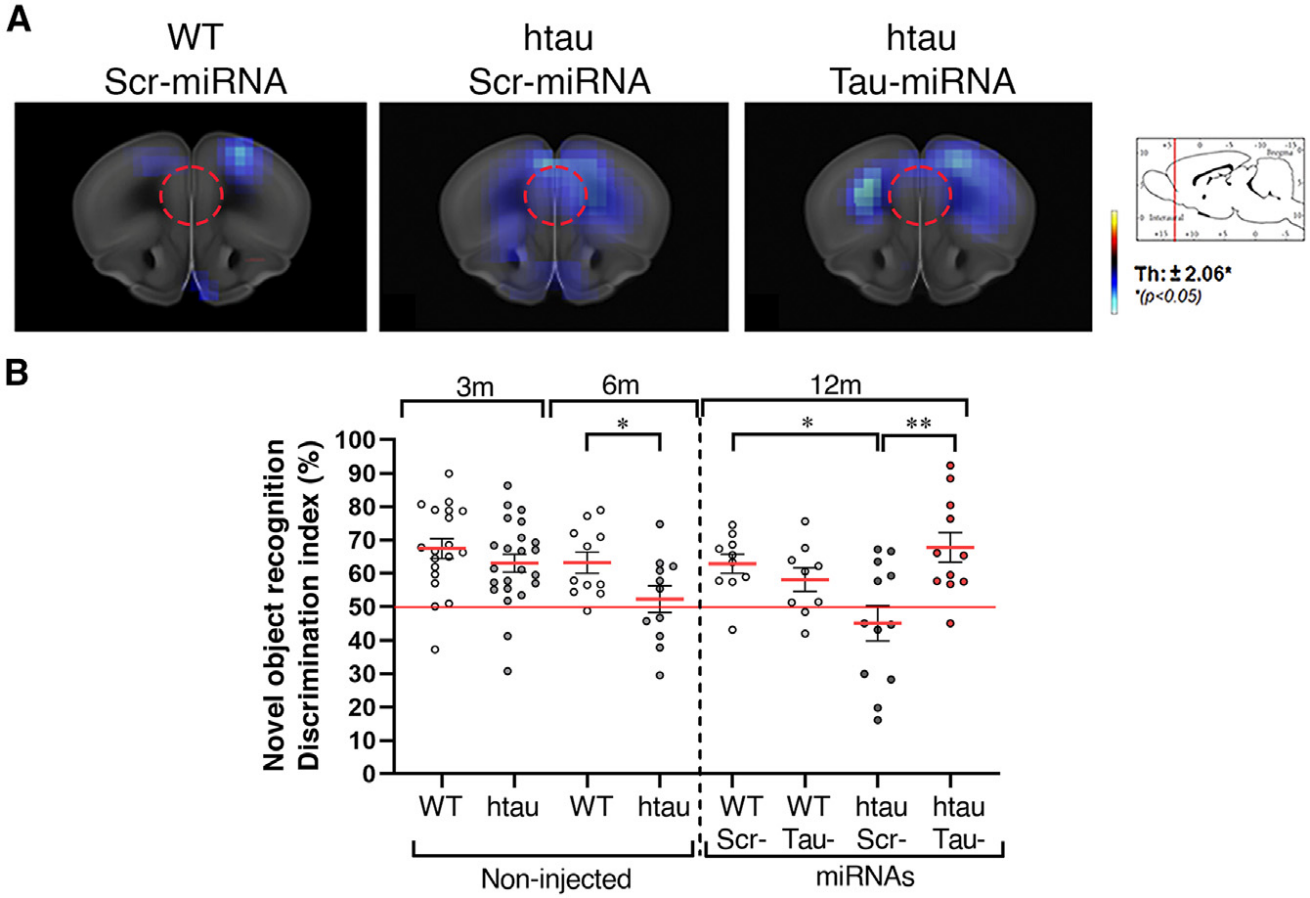
Tau reduction in the mPFC prevents glucose uptake changes and cognitive deficits in aged htau mice (A) Glucose uptake in miRNA-injected mice determined by 18F-FDG-PET imaging. Images show the comparison within each group between 6 and 12 months of age (WT Scr n = 6, htau Scr n = 6, htau Tau n = 8). Color represents a statistical change (∗p < 0.05, unpaired t test). Red-yellow look-up table (right) indicates 18F-FDG uptake increases, while blue indicates uptake decrease. The red dotted circles indicate the miRNA injection area. (B) Discrimination index in the NOR test in non-injected (left) and miRNA-injected mice (right). Non-injected: 3m WT n = 19, htau n = 22; 6m WT n = 11, htau n = 11. miRNA injected: WT Scr n = 10, WT Tau n = 9, htau Scr n = 12, htau Tau n = 11; ∗p < 0.05, ∗∗p < 0.01, 3m and 6m unpaired t test and 12m one-way ANOVA followed by Tukey’s *post hoc* test. Data are shown as scatter dot plots, with mean ± SEM. See also Figure S4.

Considering the biochemical, anatomical outcomes, and electrophysiological outcomes observed upon tau reduction, we reasoned that tau knockdown could prevent tauopathy phenotypes related to prefrontal neurodegeneration. We first conducted *in vivo* microPET scans using 18F-fluorodeoxyglucose positron emission tomography ([18-F]-FDG-PET to measure glucose uptake as a readout of neuronal degeneration, a routine diagnosis method used in patients with tauopathies. Brain images were obtained by [18-F]-FDG-PET at 6 and 12 months, and differences in glucose uptake during aging were calculated (Figure 6; in each panel blue indicates significant reduction in FDG uptake in mice at 12 months old compared to 6 months old). WT mice showed slight metabolic changes in cortical areas but not in the mPFC (Figure 6A, left panel), whereas control htau mice showed a more significant decrease in glucose uptake, particularly in the prefrontal cortex (Figure 6A, middle panel). However, htau mice injected with Tau-miRNA did not show reduction in FDG uptake in the mPFC (Figure 6A, right panel), indicating that tau reduction prevented glucose uptake deficits during aging. Finally, to obtain a behavioral readout of cognitive performance in miRNAs treated htau mice, we used the novel object recognition (NOR) task. Our previous studies described that htau mice present impairments in object recognition from 6 to 12 months of age.^44^ Consistently, here we observed that htau mice showed normal object discrimination at 3 months old but a severe impairment in object recognition at 6 and 12 months of age (Figure 6B). However, a discrimination index similar to WT mice was observed at 12 months old in Tau-miRNA-injected htau mice (Figure 6B), while the Scr-miRNA htau group were unable to discriminate between objects. These results indicate that Tau-miRNA expression in the mPFC prevented the impairment in the NOR task in aged htau mice. It is noteworthy that Tau-miRNA treatment did not affect other behavioral phenotypes of htau or WT mice (Figures S4F‒S4I).

## Discussion

In this study we designed artificial miRNAs that target the human *MAPT* mRNA to achieve stable reduction of tau protein synthesis, both in human-derived neurons in culture and in the mouse brain. We observed that lowering tau in normal human neurons significantly decreased their excitability but did not affect neuronal morphology or impaired axonal transport, indicating that the decrease of functional tau protein in mature neurons is not detrimental. In turn, we observed that tau knockdown *in vivo* reduces insoluble tau accumulation and cognitive decline in the htau model of tauopathy.

Although the canonical function of tau relates to microtubule stabilization, we did not observe dramatic changes neither in neuronal arborization or in differentiated human neurons expressing Tau-miRNA. Indeed, our present results suggest that tau reduction might have a facilitation effect over axonal transport, considering the increase in number of vesicles observed within the axon in Tau-miRNA-transduced neurons. We previously reported that bidirectional axonal transport was differentially regulated in human neurons in culture by changes in tau isoforms contents,^46^ and other *in vitro* experiments also showed that tau has a stronger effect in anterograde transport than on the retrograde motor dynein.^47^ Further experimental approaches might be useful to unravel if lowering tau is sufficient to facilitate axonal transport.

Remarkably, knockdown of endogenous tau in human neurons in culture dramatically reduced electrical activity, shown by a significant decrease in firing rates and currents, as well as changes in the location of the axonal initial segment. These data suggest that tau might have a physiological role in neuronal firing, beyond its function in the excitotoxicity mediated by pathology. Little is known about the mechanisms linking tau with normal excitability of neurons. Most studies so far have focused on describing tau as an enhancer of firing changes in neurological disorders, showing how tau ablation or knockdown could be protective in different mouse models of disease.^27^^,^^39^^,^^48^ Indeed, our results showed that firing rates of cortical pyramidal neurons—which are increased in aged htau mice—were recovered to WT levels in Tau-miRNA-injected mice, suggesting that the phenotypic rescue observed upon tau reduction might correlate with modulation of neuronal firing. It has been widely reported that tau mislocalization in human tau transgenic mice leads to increased neuronal excitability,^38^^,^^39^^,^^49^ which was suggested to relate to neurodegeneration.^50^^,^^51^^,^^52^ Tau-deficient mice—or those with postnatal tau silencing—showed protection over excitotoxic brain damage in several models.^48^^,^^53^^,^^54^^,^^55^^,^^56^ In addition, our current findings revealing that local miRNA injection into the mPFC reduces pyramidal neuron—but not interneuron—firing rates is also consistent with previous reports demonstrating that pathological tau differentially affects glutamatergic versus GABAergic neurons.^39^^,^^51^^,^^57^ Based on our results in neurons in culture, one might hypothesize that global reduction of physiological tau in the brain might have a harmful impact on neuronal firing beyond affected neurons; however, considering the evidence observed in tauopathy models, we can speculate that such side effects could be minimized if knockdown were targeted only to vulnerable neurons accumulating pathological tau into specific brain areas.

We also report here that local miRNA delivery into the mPFC of htau mice prevented age-dependent pathological tau accumulation and cognitive decline. It is noteworthy that the reduction of tau protein achieved *in vivo* by Tau-miRNA injection in the PFC was only about 30% (Figure 3C), being much lower than the 75% decay observed in human neurons in culture (Figure 1G). However, this modest reduction in the PFC was enough to prevent the accumulation of insoluble tau (Figure 3E) and hyperphosphorylated tau clusters at presynaptic terminals (Figure 4), two main readouts of pathological tau. Moreover, we previously observed in FDG-PET scans that aging htau mice show a significant decrease in glucose uptake in the mPFC,^44^ which correlates with neurodegeneration, as seen in patients with tauopathy.^58^ Here, we found that such metabolic change was prevented by Tau-miRNA injection, restricted to the targeted area without altering glucose uptake in other brain structures. Finally, a recovery in cognitive decline was also observed after tau reduction in the mPFC. The htau mouse is described as a model of mild-cognitive impairment^42^ and we previously described that the mPFC is one of the main structures affected in these mice, so we used an NOR task paradigm that relies on proper PFC function (working memory and executive function).^43^^,^^59^ However, even though the PFC is a vulnerable region in tauopathies such as frontotemporal lobar degeneration (tau-FTLD) and AD, additional studies targeting other affected brain areas in different animal models will provide stronger evidence about the potential use of Tau-miRNAs.

Targeting abnormal tau protein accumulation is undoubtedly the most promising therapeutic strategy underway for tauopathies,^24^^,^^27^^,^^60^^,^^61^ including AD. Tau reduction seems to be well tolerated in humans, with clinical trials showing so far that ASOs do not lead to overt structural or functional changes in the brain.^62^ However, the major constraint in therapeutic approaches based on tau downregulation would be the deletion of normal tau, with its physiological properties, instead of just deleting the pathological forms. To overcome this issue, therapeutic approaches can be refined to target only affected brain nuclei and vulnerable neurons. In this sense, designed miRNAs hold several advantages: (1) unlike small-molecule drugs, which often have pleiotropic effects, artificial miRNAs allow more precise targeting, as they can be locally delivered by viral vectors, without altering gene expression in areas not affected by disease^63^^,^^64^; (2) in contrast to short hairpin RNAs (shRNAs), which are ubiquitously expressed under Pol III promoters, the expression of miRNAs can be achieved under Pol II promoters, allowing their expression to be targeted to specific cell types^64^; (3) artificial miRNAs enter the miRNA biogenesis pathway at an early stage and undergo two-step processing by the RNases DROSHA and DICER, similar to most endogenous mammalian miRNA transcripts, being less immunogenic, more efficient, and less toxic.^33^ In summary, designed miRNAs represent a promising tool for long-term repression of genes involved in neurodegeneration. In fact, a designed miRNA that target huntingtin^65^ is currently being tested in a clinical trial for Huntington’s disease.^64^^,^^65^

The main translational limitation with this therapeutic approach would be the expression of miRNAs by viral vectors, which involves invasive delivery into the brain. Nevertheless, this strategy would require only one injection to achieve stable expression. Ongoing clinical trials show that single brain injections of viral vectors are well tolerated and yield clinical benefits in patients with neurological disorders,^66^^,^^67^ including an LV-based ongoing trial for Parkinson’s disease.^68^ It is noteworthy that, in this study, we chose LVs to express the engineered miRNAs to restrict the expression to the PFC. LVs allow a stable, long-term expression and, due to their limited spreading within the brain, they would not transduce off-target areas. In addition, we used a low multiplicity of infection (MOI ≈ 5) to avoid a massive overexpression of the artificial miRNAs in the transduced area and prevent potential side effects due to a dramatic reduction in tau levels. However, for clinical trials, non-integrative AAVs might be safer delivery method and would allow wider areas to be targeted with a single injection.

During the last 20 years, gene therapy strategies have reached a momentum in translational studies and clinical trials, thanks to development of more efficient delivery agents and, particularly in the last decade, with the emergence of gene edition technology.^66^ However, some DNA targeting interventions are still difficult to achieve and even more difficult to revert, leading to bottlenecks in developing effective and safe therapies. In this scenario, RNA therapies represent a plausible alternative.^69^^,^^70^ Unlike gene editing, RNA therapies do not alter the actual sequence of a mutated gene but instead alter its output. Those changes are temporary and more versatile, as they represent a solution for diseases that arise from changes in either RNA transcription, processing, or turnover. Therefore, RNA-based therapies seem to open up limitless possibilities to treat all categories of diseases.^71^

We provide here proof of concept about target engagement for artificial miRNAs to induce long-term tau reduction in human neurons *in vitro* and in a tauopathy model *in vivo*, leading to significant improvement in biochemical and phenotypic markers of tau pathology. Altogether, our data suggest that early local administration of Tau-miRNAs into vulnerable nuclei might be a plausible disease-modifying therapy for tauopathies. However, further studies in preclinical models are necessary to verify the efficiency and safety of this strategy and set the grounds to develop tailored therapies for tauopathies.

## Materials and methods

### miRNA design

Artificial miRNAs were designed following an in-house combination of described rules and a free access algorithm from the Whitehead Institute for Biomedical Research, MIT: http://sirna.wi.mit.edu. We combined the endogenous miR-155 backbone retaining native flanking sequences with designed siRNA sequences to target *MAPT* transcript. First, five siRNA sequences were obtained combining published design methods.^72^^,^^73^ Briefly, duplex sequence consensus was N2[CG]N8[AUT]N8[AUT]N2, avoiding successive 4-nt repeated sequences of the same base, with GC content in the sequence between 45% and 50%. Target regions were chosen at the exon 2/3 junction (alternative exons) and at exon 11 (constitutive exon) to maximize silencing. Thermodynamic values of the duplex were calculated according to the energy at the 5′ end of the sense strand (Es) and the energy of the 5′ end on the strand considered antisense (Eas). Only Eas < Es were selected with ΔT values between −5 and −3. Five initial sequences were obtained targeting the human *MAPT* transcript, of which two were selected after BLAST against human and mouse (sequences with >17 nt off-target match were discarded). This conservative selection reduces the off-target effect of miRNAS^32^^,^^33^ The Scr sequence was obtained with the same GC content with less than 15-nt match obtained after BLAST. Selected siRNA sequences were embedded into a miRNA backbone containing the arms of miR-155 followed by the antisense sequence, the loop, and the sense sequence. From the siRNA sense sequence, nt 10 and 11 were removed to allow the formation of a 3D structure that optimizes the binding with the RNA-induced silencing complex (RISC) system.^72^

Full sequences of the artificial miRNAs used in this study are as follows: [miRNA5′arm -Antisense(21nt) - *Loop*-sense (19nt)- miRNA 3′arm].

#### Tau-miRNA 166 (target E2/3)

ACCGGTGTCGACTTTAAAGGGAGGTAGTGAGTGGACCAGTGGATCCTGGAGGCTTGCTGAAGGCTGTATGCTGAATGCCTGCTTCTTCAGCTTT*GTTTTGGCCACTGACTGAC*AAAGCTGAAAGCAGGCATTCAGGACACAAGGCCTGTTACTAGCACTCACATGGAACAAATGGCCCAGATCTGGCCGCACTCGAGATATCTAGAATTCACTAGTGAGCTC.

#### Tau-miRNA 724 (target E11)

ACCGGTGTCGACTTTAAAGGGAGGTAGTGAGTGGACCAGTGGATCCTGGAGGCTTGCTGAAGGCTGTATGCTGTAATGAGCCACACTTGGAGGT*GTTTTGGCCACTGACTGAC*ACCTCCAAGTGGCTCATTACAGGACACAAGGCCTGTTACTAGCACTCACATGGAACAAATGGCCCAGATCTGGCCGCACTCGAGATATCTAGAATTCACTAGTGAGCTC.

#### Scr-miRNA

accggtGTCGACTTTAAAGGGAGGTAGTGAGTGGACCAGTGGATCCTGGAGGCTTGCTGAAGGCTGTATGCTGAAATGTACTGCGCGTGGAGAC***GTTTTGGCCACTGACTGAC***GTCTCCACGCAGTACATTT**CAGGA**CACAAGGCCTGTTACTAGCACTCACATGGAACAAATGGCCCAGATCTGGCCGCACTCGAGATATCTAGAATTCACTAGTGAGCTC.

### LVs

Artificial miRNAs were subcloned under the human synapsin promoter between AgeI and EcoRI sites into a LV backbone previously described.^34^^,^^43^^,^^45^^,^^74^ Lentiviral particles were generated as previously described.^43^^,^^75^ Briefly, HEK-293T cells were grown on DMEM, supplemented with 10% (v/v) fetal bovine serum (FBS; Natocor, Argentina), 0.5 mM L-glutamine, 100 U/mL penicillin, and 100 μg/mL streptomycin (Thermo Fisher). Cells at 80%–85% confluence were co-transfected with a lentiviral shuttle vector (Tau-miRNA 166, Tau-miRNA 724, or Scr-miRNA) together with helper vectors encoding packaging and envelope proteins (cytomegalovirus [CMV] Δ8.9 and CMV-vesicular stomatitis virus G protein [VSVg], respectively). Viral particles were harvested from the culture medium 36 h after transfection and treated with RNase-free DNase I (Thermo Fisher). Viral vectors were purified by centrifugation and filtering (45-μm pore), concentrated by ultracentrifugation at 100,000 × *g* (Ti 90 rotor, Beckman), and resuspended in sterile PBS. After performing titration, 10-μL aliquots of viral particles were stored at −80°C.

### Screening Tau-miRNAs in cultured cells

SH-SY5Y cells (ATCC CRL-2266; passage #12–14) were cultured in a 24-well plate (0.2 × 10^6^ cells) and grown as a monolayer on DMEM/F12 medium, supplemented with 10% (v/v) FBS (Natocor, Argentina), 0.5 mM L-glutamine, 100 U/mL penicillin, and 100 μg/mL streptomycin (Thermo Fisher). When reaching 85% confluency, cells were transduced with LVs containing Scr-miRNA, Tau-miRNA 166, Tau-miRNA 724, or an equimolar combination of both Tau-miRNA 166 and Tau-miRNA 724 (Tau-miRNAs 166 + 724; 1:1). Non-transduced cells were used as a negative control. Three weeks post transduction, cells were processed to obtain protein for western blot analysis. From the results obtained (see Figure S1A), consecutive treatments with human-derived neurons or injections in mice were defined as Scr-miRNA (control) or Tau-miRNA (1:1 mix of Tau-miRNA 166 and Tau-miRNA 724, to obtain maximum efficiency of silencing).

### hiPSC culture and neuronal differentiation

Neurons were derived from hiPSCs as previously described.^37^ Briefly, irradiated murine embryonic fibroblasts (MEFs) were plated over gelatin-coated Petri dishes 24 h before hiPSC plating and maintained in DMEM complete (high-glucose DMEM, 10% FBS, 1% Glutamax, 1% penicillin-streptomycin; Thermo Fisher). hiPSCs were grown at 37°C, 95% humidity, and 5% CO_2_ in HES medium (KO DMEM, 20% KO serum replacement, 1% Glutamax, 1% non-essential amino acids, 0.1% beta-mercaptoethanol, 4 ng/mL bFGF; Thermo Fisher) to allow colony formation. When colonies reached optimal size, they were first transferred to a Petri dish and, after 12–18 h, to 25-cm^2^ flasks and grown in suspension to allow embryoid body formation, which was induced by neural induction medium (NIM; DMEM/F12, 1% N2 supplement, 1% non-essential amino acids, 280 UI/mL heparin, and 1% penicillin-streptomycin; Thermo Fisher). Embryoid bodies were then transferred to laminin-coated six-well plates for neural rosette formation. After 7–14 days of growth, the neural rosettes were picked and transferred to 25-cm^2^ flasks and maintained in NIM complete medium (2% B27 supplement, 0.1% ascorbic acid; Thermo Fisher) for up to 1 month, changing the growth medium every 2 days. Neural rosettes were picked and dissociated by an 8 min of acutase and trypsin treatment. The reaction was blocked by trypsin inhibitor (Thermo Fisher) and the suspension centrifuged for 5 min at 1,000 rpm. The pellet was washed with DMEM/F12, disaggregated to single cell, and resuspended in neural differentiation medium (NDM; Neurobasal, 1% N2 supplement, 2% B27 supplement, and 1% penicillin-streptomycin; Thermo Fisher). Approximately 3 × 10^4^ cells were plated on glass-coated coverslips in 24-well plates precoated with 0.1 mg/mL poly-ornithine (Sigma-Aldrich) and 20 μg/mL laminin (Thermo Fisher). Neuronal cultures were then maintained in 500 μL/well of NDM complete medium (NDMc; laminin, cAMP, ascorbic acid, 10 ng/mL BDNF, 10 ng/mL GDNF; recombinant, Thermo Fisher). Plated neurons were maintained in culture, with half of the medium changed every 3 days. Initially, approximately 40% confluency was intended, allowing for subsequent coverage due to extensive neurite projection extension.

Neuronal identity was verified by immunohistochemistry, morphological, and functional analyses (see below). Neurons were washed with PBS and fixed with 4% paraformaldehyde and 4% sucrose in PBS for 30 min at 37 °C at DIV14. After fixation, cells were washed twice with PBS for 10 min and permeabilized with 0.1% Triton X-100 for 10 min at room temperature. Cells were incubated at room temperature for 1 h using a blocking solution consisting of 3% BSA, 0.1% Triton X-100, and 10% goat serum in PBS. Cells were then stained with primary antibodies in blocking solution and incubated overnight at 4°C. Cells were then rinsed in PBS and stained with secondary antibodies at room temperature for 2 h, stained for 30 min with DAPI, and mounted on slides with MOWIOL (Calbiochem). Primary antibodies used were as follows: anti-Nestin (1:200; rabbit; Sigma-Aldrich), anti βIII-tubulin (1:1,000; mouse; BioLegend), anti-p-tau (1:200; rabbit; Calbiochem), and secondary antibodies against mouse and rabbit IgG conjugated to Alexa Fluor 564 or Alexa Fluor 488 (1:500). Fixed cells were imaged with an inverted Zeiss LSM 780 confocal microscope using an oil-immersion objective (40×/0.55 NA). All neurons used in this study showed neuronal differentiation and well-defined polarization (see Figure S1B).

### Transduction and transfection of hiPSC-derived neurons

On DIV14, neurons were transduced with LVs containing miRNAs in an MOI between 5 and 10, as described previously.^34^ After 12 h, cells were topped up with 300 μL of fresh NDMc medium. Twenty-one days after LV transduction (on DIV35), neurons were transfected with 1 μg of pcDNA3-APP-YFP in a transfection mixture of OptiMEM and Lipofectamine 2000, as previously described.^34^ Two hours after transfection, the culture medium was replaced and, 48 h later (on DIV37), neurons were analyzed by live-cell imaging for transport analysis and patch-clamp recordings, fixed for Sholl analysis, or processed to obtain RNA and protein for RT-qPCR and western blot analyses, respectively.

LV transduction was confirmed by expression of dsRed fluorescent protein at DIV35 and plasmid transfection was confirmed by expression of APP-YFP fusion fluorescent protein at DIV37. Cells were imaged with an inverted Zeiss LSM 780 confocal microscope using an oil-immersion objective (40×/0.55 NA).

### RNA isolation from neurons in culture and detection of total tau mRNA

Total RNA was isolated from hiPSC-derived neurons using the AllPrep DNA/RNA mini kit (Qiagen) from high-density cultures at DIV37. RNA quality was evaluated by measuring the absorbance for 260/280 and 260/230 ratios (NanoDrop, Thermo Fisher) and by revealing 28S and 18S RNA bands (without genomic DNA band) in a 2% agarose Rnase-free gel with ethidium bromide. Reverse transcription was performed with 0.5 μg of RNA with the TaqMan RT kit (Applied Biosystems) in a total volume of 10 μL, with an equimolar ratio of oligo (dT) and random hexamers. Reverse transcription conditions were: 10 min at 25°C, 30 min at 48°C, and a final step of 5 min at 95°C. To perform the relative quantification of total tau by real-time PCR, specific pairs of primers were used to amplify all six human tau mRNA transcripts isoforms, and were designed to target different exons to avoid DNA amplification: forward E7, 5′-AGCCAAGACATCCACACGTT-3′ and reverse E8, 5′-ATCAGAGGGTCTGAGCTACCA-3′. For normalization, *GAPDH* mRNA was detected using the following sequence of primers: 5′-GGTCTCCTCTGACTTCAACA-3′ (forward) and 5′-GTGAGGGTCTCTCTCTTCCT-3′ (reverse). qPCR reactions were performed in triplicate with 25 ng of cDNA and 5 μL of Power SYBR Green PCR Master Mix (Applied Biosystems) in a final volume of 10 μL using an MJ Research Opticon 2 real-time PCR thermal cycler under the following cycling conditions: after initial denaturation at 95°C (10 min), 39 cycles at 95°C (10 s), the primer-specific annealing temperature was 58°C (30 s), and elongation at 72°C (45 s). Data were analyzed with the Opticon monitor 3 software (Bio-Rad) to obtain the ΔCT per sample. Values for total tau mRNA contents were normalized to the *GAPDH* reference gene.

### Sholl analysis

Semi-automated Sholl analysis was done using the Simple Neurite Tracer (SNT) tool from the Neuroanatomy plugin in ImageJ.^76^ Neurons were fluorescently labeled with MAP2 (1:300; rabbit polyclonal; Santa Cruz Biotechnology) using the fixation and immunofluorescence protocol as described above. Eight-bit images were used to determine dendrites and branches, which were semi-automatically traced with SNT. After tracing, Sholl analysis was performed with the Neuroanatomy tool, where a series of concentric consecutive rings from the soma were positioned evenly spaced to cover the extent of the projections. This radius step size of the rings was kept constant for all images. Each projection that intersected with a ring was counted, contributing to a cumulative count per ring. A peaked distribution was observed at the initial rings, reflecting an increased projection arborization near the cell body. As the rings moved further from the soma center, the number of intersecting projections decreased.^76^

### Live-cell-imaging and axonal transport analysis

Imaging of live cells and kymograph analysis of axonal transport was performed as described previously.^37^ Briefly, 30 s videos of APP-YFP moving particles in neurons were recorded using an inverted epifluorescence microscope (Olympus IX81) connected to a charge-coupled device (CCD) camera (Olympus DP71/12.5 megapixels). Cultures were observed under a 100× lens (1.45 NA) and maintained at 37°C, 5% CO_2_, and 10% humidity using a CO_2_ humid chamber and heated stage (Tokai, Japan). Directionality was determined by tracking fluorescent axons. To avoid introducing biases due to the gradient concentration of tau in axons, imaging was performed in axons at their middle part separated by at least two fields of view distance (∼200 μm) from cell bodies and from axonal tips. Kymographs were generated from the recordings with ImageJ using the Multiple Kymograph plugin. Kymographs were plotted and vesicle directionality and density were extracted for analysis using custom-made MATLAB routines.^37^

### Electrophysiological recordings of neurons in culture

For electrophysiological recordings, cultured hiPSC-derived neurons were perfused in artificial cerebrospinal fluid (ACSF; mM): 125 NaCl, 2.5 KCl, 2.3 NaH_2_PO_4_, 25 NaHCO_3_, 2 CaCl_2_, 1.3 MgCl_2_, 1.3 Na^+^-ascorbate, 3.1 Na^+^-pyruvate, and 10 dextrose (315 mOsm) and bubbled with 95% O_2_/5% CO_2_. Whole-cell recordings were performed using microelectrodes (6–10 MΩ) filled with (in mM) 120 potassium gluconate, 4 MgCl_2_, 10 HEPES buffer, 0.1 EGTA, 5 NaCl, 20 KCl, 4 ATP-tris, 0.3 GTP-tris, and 10 phosphocreatine (pH 7.3; 290 mOsm). Spiking was assessed keeping the resting membrane potential at −70 mV in current clamp and passing successive depolarizing current steps of 10-pA increment and 500-ms duration. Voltage-dependent Na^+^ and K^+^ currents were measured in voltage clamp after leak subtraction using a p/-6 protocol and detection of the fast inward peak for Na and the late outward plateau for K. Input resistance was obtained from current traces evoked by a hyperpolarizing step of 10 mV. Series resistance was typically 10–20 MΩ, and experiments were discarded if higher than 50 MΩ. Recordings were obtained using Multiclamp 700B amplifiers, (Molecular Devices, Sunnyvale, CA), digitized, and acquired at 20 kHz onto a personal computer using the pClamp10 software.

### AIS morphology

Cells were fixed and immunolabeled using the same protocol for checking quality of hiPSC-derived neurons. In this assay, cells were stained with primary antibody anti-ankyrin-G (1:200; mouse monoclonal; Neuromab) and with secondary antibody anti-mouse IgG conjugated to Alexa Fluor 488 (1:500). Two parameters that describe the AIS morphology were evaluated, the AIS length and the distance to soma. Briefly, the AIS length was estimated by measuring the length of ankyrin G staining of single axons, and the distance to soma was assessed as the distance from the soma to the beginning of the AnkG staining. Images were imported in ImageJ and pixels were converted into micrometers.

### Mice and experimental design

All animal procedures were designed in accordance with the NIH Guidelines for the Care and Use of Laboratory Animals. Protocols were approved by the Institutional Animal Care and Use Committee of INGEBI-CONICET and University of Buenos Aires. Mice were housed in standard conditions under 12-h dark/light cycle with *ad libitum* access to food and water. Htau transgenic mice,^41^ in a C57BL/6J background, were obtained from Jackson Laboratories (Bar Harbor, ME, United States; B6.Cg-Mapt^tm1(EGFP)Klt^Tg(MAPT)8cPdav/J. Strain number: 005491) and bred in house. To confirm the presence of the human *MAPT* transgene and the mouse *Mapt*^−/−^ background, all mice used in this study were genotyped by PCR as previously described.^43^^,^^45^^,^^75^ Htau mice were in-house backcrossed to C5BL/6J mice every 12 generations to refresh breeders.

Experimental groups (htau or WT littermates; see Table S1) were randomly allocated to receive either Tau-miRNA (166 + 724; 1:1) or Scr-miRNA. At 12 months of age, *in vivo* analyses were performed, consisting of behavioral tests, positron emission tomography, and electrophysiological recordings. Biochemical analyses were performed *postmortem* as indicated later in this section. For protein extraction, mice were sacrificed by cervical dislocation and the injected area was dissected and stored at −80°C until use. For immunofluorescence array tomography analyses, mice were perfused transcardially with 4% paraformaldehyde in 0.1 M phosphate buffer (pH 7.4).

### Stereotaxic injections

LVs were delivered into the mPFC as previously described.^43^ Briefly, mice (males and females) aged 8–10 weeks (weight 25–30 g) were anesthetized with isoflurane 0.5%–2% (2% for induction/0.5%–1% for maintenance, Baxter) in medical grade oxygen with an air flow at 2.5 L min^−1^ and placed into a stereotactic frame (Stoelting CO). A 10-μL Hamilton syringe coupled to a 36G stainless steel tube (Cooper Needleworks, United Kingdom) was used to inject 1.5 μL of lentiviral suspension (0.5 ×10^7^ TU/mL; 0.2 μL/min) per site of injection, bilaterally, at four sites into the mPFC, following coordinates of mouse atlas (Paxinos and Franklin, 2013) (in mm): AP = +2.3, LM = ±0.5, DV = −1.8, and −2.2. Immediately after surgery, mice received analgesic (Aplonal; 1 mg/kg, subcutaneously [s.c.]), repeated 24 h later. Any animal showing signs of pain or discomfort after surgery was sacrificed following the endpoint protocol.

### Protein extraction and western blotting

Total protein was collected from neurons at DIV37 or from SH-SY5Y cells, in lysis buffer containing 40 mM Tris-HCl (pH 7.5), 150 mM NaCl, 1% Igepal, and 1× protease inhibitor cocktail (Sigma-Aldrich) and centrifuged for 10 min at 10,000 rpm at 4°C. For protein extraction from mouse brain, the PFC and motor cortex 1 (M1) were dissected and homogenized with a buffer containing 50 mM Tris-HCl (pH 7.4), 150 mM NaCl, 2 mM EGTA, and protease and phosphatase inhibitor cocktail (Thermo Fisher). After homogenization with a motorized tissue grinder, protein extracts were centrifuged at 13,500 rpm for 15 min at 4°C.

Equal amounts of total protein (determined with Pierce BCA Protein Assay Kit, Thermo Fisher) were separated on 10%–12% SDS-polyacrylamide gels (prepared with acrylamide and N,N′-methylenebisacrylamide 30%) and transferred using a semi-dry transfer system to nitrocellulose membranes (Bio-Rad). See-Blue Plus 2 (Thermo Fisher) was used as a molecular-weight marker. Membranes were blocked in 5% (w/v) non-fat dry milk (La Serenisima, Argentina), 0.05% v/v Tween 20 in TBS for 1 h at room temperature. Primary antibodies were used in diluted blocking solution to incubate blots overnight at 4°C: anti-total tau (1:10,000; rabbit polyclonal; Dako, Denmark) and anti-β-actin-HRP conjugated (1:10,000; mouse monoclonal; Sigma-Aldrich). After washing three times in TBS containing 0.05% v/v Tween 20, blots were incubated with secondary antibody goat anti-rabbit-HRP conjugated (1:2,000; Thermo Fisher) for 2 h at room temperature. Proteins were visualized using enhanced chemiluminescence (ECL) reagent (Thermo Fisher) exposing membranes on the GenegnomeXRQ (Syngene). Optical density was quantified using FluorChem software (Alpha Innotech) and total tau contents were normalized to actin, used as a loading control.

### Sarkosyl insolubility assay

Fractionation of insoluble proteins was performed using 250 μg of mPFC protein extract, which was incubated with 1% sarkosyl reagent (Sigma-Aldrich) for 1 h in minimal agitation at room temperature, following protocol previously reported.^43^ Protein extract with 1% of sarkosyl reagent was ultracentrifuged at 39,000 rpm (1 h) at 20°C to obtain the pellet and then washed with sarkosyl 1% by ultracentrifugation in the same conditions for 15 min. Pellet was resuspended in buffer O+^77^ for 1 h at room temperature for western blotting.

### Positron emission tomography

*In vivo* brain activity was analyzed using tracer fluorinated glucose analog (18F-FDG), which localizes in metabolically active tissues and accumulates in an activity-dependent manner. Animals were starved for 4 h and then injected with 25 μCi/gr of 18F-FDG intraperitoneally (i.p.) and left undisturbed in an individual temperature-controlled (29°C) cage for 30 min during radiopharmaceutical incorporation. Mice were then anesthetized using a mixture of isoflurane and O_2_ (4.5% for induction/1.5% for maintenance) and maintained in a warm table (35°C) during the acquisition. Images were acquired using a preclinical PET TriFoil Lab-PET 4 (3.75-cm axial length) with a dual layer of LYSO and GSO crystals, assembled in phoswich pairs. Signal readout was based on an APD-Detection (Avalanche PhotoDiode). Image reconstruction was performed on emission data through 3D ordered subset expectation maximization (OSEM) iterative reconstruction (30 iterations). All images were co-registered and normalized to a 18F-FDG template. The quantitative brain image of each mouse was normalized to the total cortex to avoid bias in the analysis. Intensity normalization was considered as a regressor variable for each factor using all-brain mean scaling (ANCOVA). Results are shown using a color scale representing a statistical parametric comparison between the groups, using the unpaired t test (p < 0.05).

### *In vivo* electrophysiological recordings

#### Data acquisition

Electrodes for extracellular recording were made as previously described.^45^ Mice were deeply anesthetized with isoflurane (2% for induction, and 0.5%–1% for maintenance, Baxter) and placed into a stereotaxic frame. The skull was exposed to clearly locate bregma. A craniotomy was performed over the mPFC coordinates (AP = +2.1 mm, LM = ±0.5 mm, bregma as reference). The tetrodes were lowered inside the brain at a speed rate of 10–20 μm/s. Stable spontaneous action potentials were sought up between −1 and −2.5 mm from the surface. Electrophysiological data were recorded at different positions (up to seven recordings were acquired per animal), with durations ranging from 15 to 30 min each.

#### Data processing and analysis

Analysis and statistical tests were implemented in MATLAB (The MathWorks, USA). Raw signals were band filtered between 300 and 6,000 Hz. Spike sorting was performed as follows. An automatic threshold was set at five times the standard deviation above the mean to detect the spike events. Detected spikes were partitioned into many clusters with a *k*-means method and then were aggregated according to their interface energy for each pair. Clusters were manually split and merged according to their principal components for the subsequent analysis. Single units were classified into two groups based on their mean spike width or waveform, measured as the time from the trough to the next peak of the mean action potential. Units with a valley to peak (y axis in the figure) greater than 440 μs were considered as putative pyramidal neurons (Figure S4A; red dots) and otherwise as putative interneurons (Figure S4A; blue dots). In this way, we distinguished neurons based on their average spike waveform, regardless of their firing rate.

Rasters at 1-ms resolution containing a sequence of zeros (no spike event) and ones (spike event) were constructed for each isolated unit. On these rasters, the firing rate (spikes per second) and the ISIs were computed for each neuron. To measure the degree of burstiness in the firing of a given neuron, an autocorrelogram with time shifts ranging from 0 to +50 ms was computed from the rasters. Next, the time shift Δt at which half the accumulated autocorrelogram value was reached. Burstiness index was defined as: $BI=\frac{\left(50\;ms\;-\;\Delta t\right)}{50\;ms}\text{.}$^78^

### High-resolution immunofluorescent array tomography

After transcardiac perfusion of mice, brains were post-fixed in 4% paraformaldehyde overnight at 4°C, incubated in 15% sucrose for 24 h, and then incubated in 30% sucrose for another 24 h. Tissue blocks containing the mPFC and the AI were cut to 300-μm-thick sections using a vibratome and then processed for array tomography as previously described,^79^ using LRWhite resin (medium grade, Ted Pella, USA). Embedded tissue was cut with a Jumbo Histo Diamond Knife (Diatome) in an ultramicrotome (Reichert-Jung, Germany). Series of 20–30 200-nm-thick sections were collected in ribbons onto glass coverslips and processed for immunofluorescence. Antibodies anti-p-tau (Thr231) AT180 (1:100; mouse; Thermo Fisher), anti-Synapsin-1a (1:200; rabbit; Cell Signaling Technology), anti-VGLUT1 (1:1,000; guinea pig; Millipore), and anti-VGAT (1:400; mouse; Synaptic Systems, Germany) were used. Fluorescent-conjugated secondary antibodies raised in donkey (Alexa 488, Alexa 647, and CY3, 1:100; Jackson ImmunoResearch, United Kingdom) were used. Sections were mounted on glass slides with SlowFade Gold Antifade (Life Technologies) and then imaged in a Leica DMR fluorescence microscope using a PL APO 63× NA = 1.32 oil objective and a Retiga R1 camera (Q-Imaging, United Kingdom). Serial images were aligned and converted into stacks using Fiji. A sampling mask of 120 × 120 μm in the mPFC and the AI was used for quantitative analysis using the Analyze Particles function, yielding values of puncta (for Synapsin, VGlut1 and VGAT) or clusters (for AT180) per μm.^3^

### Behavioral tests

Mice tested were sibling cohorts that were 3, 6, or 12 months old depending on the experimental design. NOR, open field, and elevated plus maze tasks were performed as described previously.^43^^,^^44^ Experiments were conducted between 13:00h and 17:00 h under dim illumination, in a separated behavioral room, where mice were transferred in advance. Recordings were analyzed by ANY-maze (Stoelting). All arenas and devices were cleaned between subjects to minimize odor cues.

## Statistical analyses

Data were analyzed with Prism GraphPad software. Datasets of each experiment were classified according to the p value obtained for the Shapiro-Wilk test, which determines normality. If the dataset passed the test (p > 0.05), the data structure was classified as normal distribution, and if the dataset did not pass the test (p < 0.05), the data structure was classified as non-normal distribution. For datasets classified as normal distribution, statistical tests used for comparing groups depended on the number of groups and independent variables used in the experiment: unpaired t test (two groups, one independent variable), one-way ANOVA test (three or more groups, one independent variable), or two-way ANOVA test (two independent variables). For one-way ANOVA, *post hoc* tests were used according to the type of comparisons between groups that were relevant for the experiment: Tukey’s *post hoc* test (for comparing the mean of each group with the mean of every other group) and Dunnett’s *post hoc* test (for comparing the mean of each group with the mean of a control group). When datasets had non-normal distribution, statistical tests used for comparing groups were the non-parametric tests: Mann-Whitney U test (comparisons between two groups, one independent variable) or two-sample Kolmogorov-Smirnov test (for comparisons of cumulative distribution of the datasets). See Table S2 for data structure, statistical test, p values, and power for each graph.

## Data and code availability

All raw datasets and videos are available upon request.

## Supplemental information

Document S1. Figures S1‒S4, Tables S1, and S2

Document S2. Article plus supplemental information
