# Supplementary material for: Tau reduction with artificial microRNAs modulates neuronal physiology and improves tauopathy phenotypes in mice
Source: Mol Ther. 2024 Feb 3;32(4):1080–95. doi: 10.1016/j.ymthe.2024.01.033 (PMC11163272; doi:10.1016/j.ymthe.2024.01.033)
Supplement: Document S1. Figures S1‒S4, Tables S1, and S2 [file mmc1.pdf]

YMTHE, Volume *32*

Supplemental Information

Tau reduction with artificial microRNAs modulates neuronal physiology and improves tauopathy phenotypes in mice

Carolina Lucía Facal, Iván Fernández Bessone, Javier Andrés Muñiz, A. Ezequiel Pereyra, Olivia Pedroncini, Indiana Páez-Paz, Ramiro Clerici-Delville, Cayetana Arnaiz, Leandro Urrutia, Germán Falasco, Carla Verónica Argañaraz, Trinidad Saez, Antonia Marin-Burgin, Mariano Soiza-Reilly, Tomás Falzone, and María Elena Avale

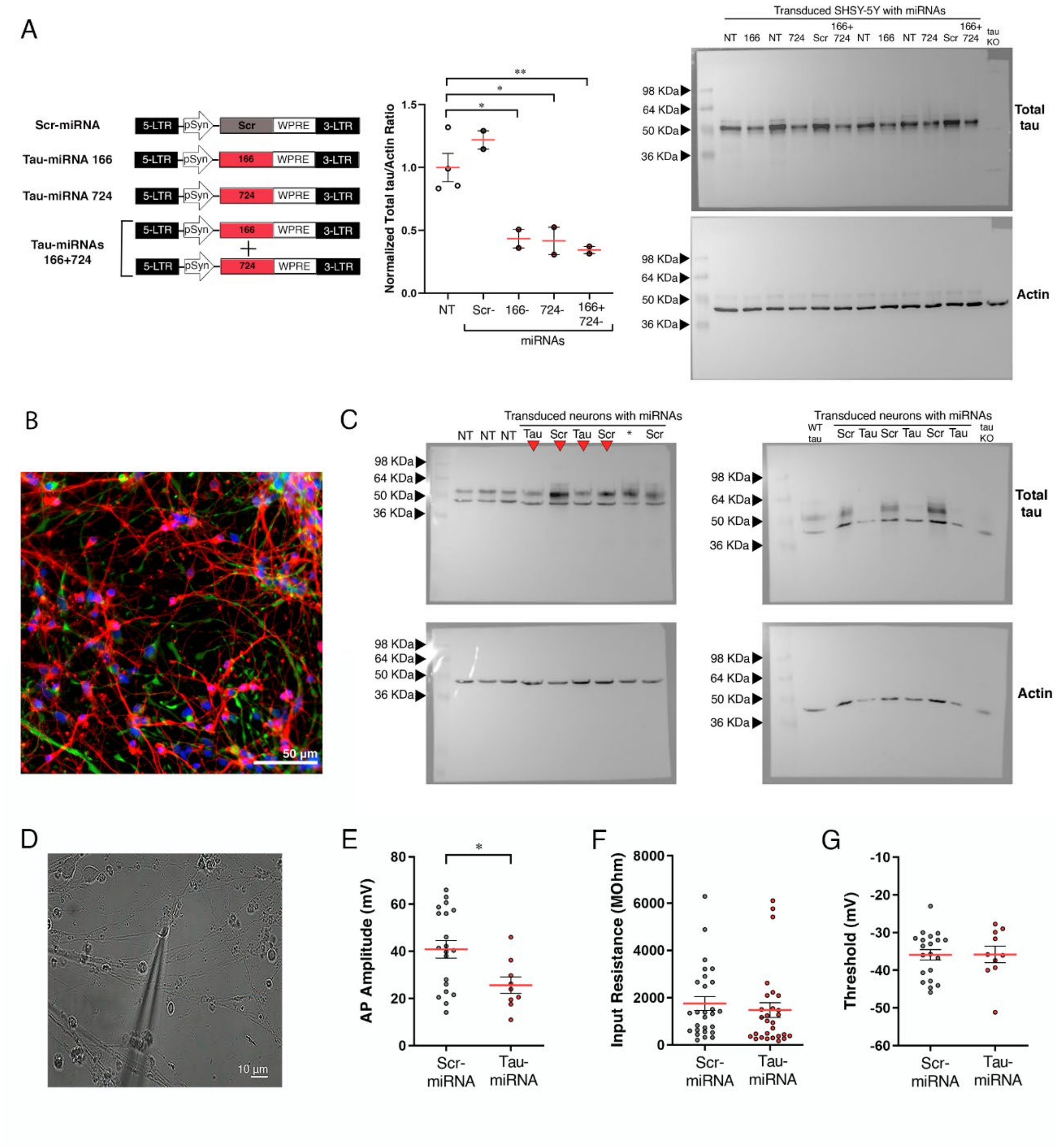

Figure S1. Tau knockdown in human differentiated neurons, related to Figures 1 and 2.

A. Screening of artificial microRNAs targeting the human *MAPT* mRNA in SH-SY5Y cells. SH-SY5Y cells were transduced with Tau-miRNA 166, Tau-miRNA 724 or with an equimolar combination of both vectors (Tau-miRNAs 166+724), and tau protein was detected by Western Blot. Cells transduced with Scr-miRNA vector or not transduced (NT) were used as controls. **Left:** Quantification of total tau protein contents normalized to actin, used as a loading control. NT n=4, Scr-miRNA n=2, Tau-miRNA 166 n=2, Tau-miRNA 724 n=2, Tau-miRNAs 166+724 n=2; **p*<0,05, ***p*<0,01, One-way ANOVA followed by Dunnett's *post hoc* test. Data is shown as scatter dot plots, with mean ± SEM. **Right:** Full bots used for quantification of total tau protein contents in SH-SY5Y cells. Tau KO was a sample obtained from brain extract used as a negative control. **B.** Immunofluorescent staining of βIII-tubulin and nestin (red and green respectively) in human differentiated neurons at DIV14. **C.** Full bots used for quantification of total tau protein contents in transduced human neurons (Scr- or Tau-miRNA). Actin was used as a loading control. Non-transduced controls (NT) were included in the blots but not in the analysis. WT tau and tau

KO were samples obtained from brain extracts used as a positive and negative controls respectively. (*) indicate outlier samples not included in the analysis. Red arrows show the samples used for the panel shown in the main section. **D.** Representative image of a patch-clamp configuration for a transduced neuron. **E-G.** Values of (**E**) the Action Potential Amplitude, (**F**) Input Resistance and (**G**) Threshold obtained for both groups. Scr-miRNA n=20, Tau-miRNA n=10; **p*<0,05, E and G: Unpaired t-test, F: Mann-Whitney U test. Data is shown as scatter dot plots, with mean ± SEM.

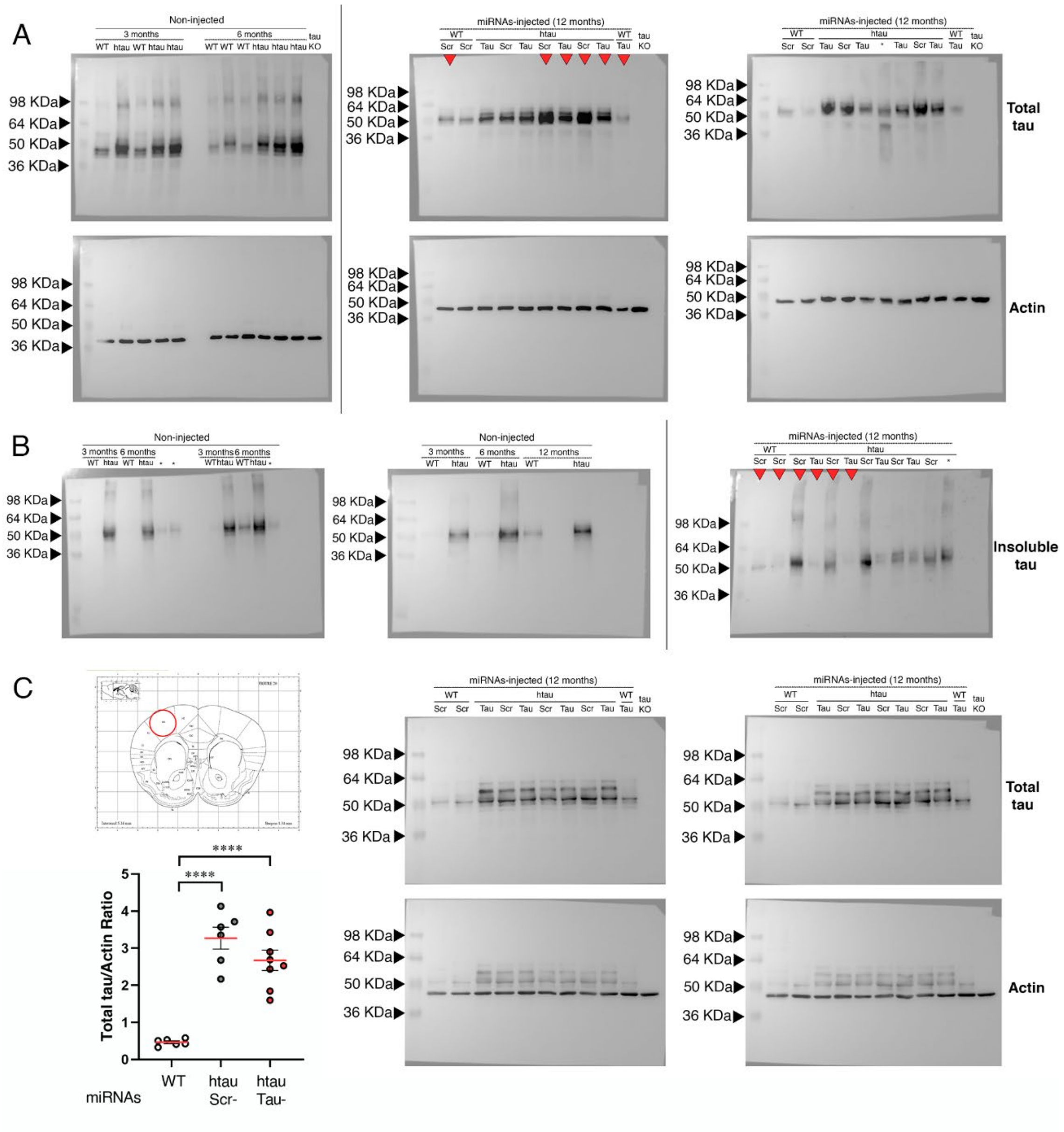

Figure S2. Reduction of total and insoluble tau contents in the medial prefrontal cortex of htau mice, related to Figure 3.

A-B. Full bots used for quantification of (**A**) total tau protein and (**B**) tau insoluble contents in the mPFC of non-injected (3- and 6-months old) and miRNAs injected (12-months-old) mice. For total tau quantification, actin was used as a loading control and tau KO as a negative control. (*) indicate outlier samples not included in the analyses. Red arrows show the samples used for the panels shown in the main section. **C.** Tau-miRNAs do not affect total tau levels in the motor cortex 1 (M1) of injected mice. **Right:** Quantification of total tau protein contents in the M1 of miRNAs injected mice,

normalized to actin as a loading control. WT n=6, htau Scr- n=6, htau Tau- n=8; ****p<0,0001, One-way ANOVA followed by Tukey's *post hoc* test. Data is shown as scatter dot plots, with mean ± SEM. WT group represents pooled samples from Scr- and Tau- miRNAs injected groups, with similar values obtained for total tau levels. **Left:** Full blots used for quantification of total tau contents in the M1 for all groups. Tau KO was used as a negative control.

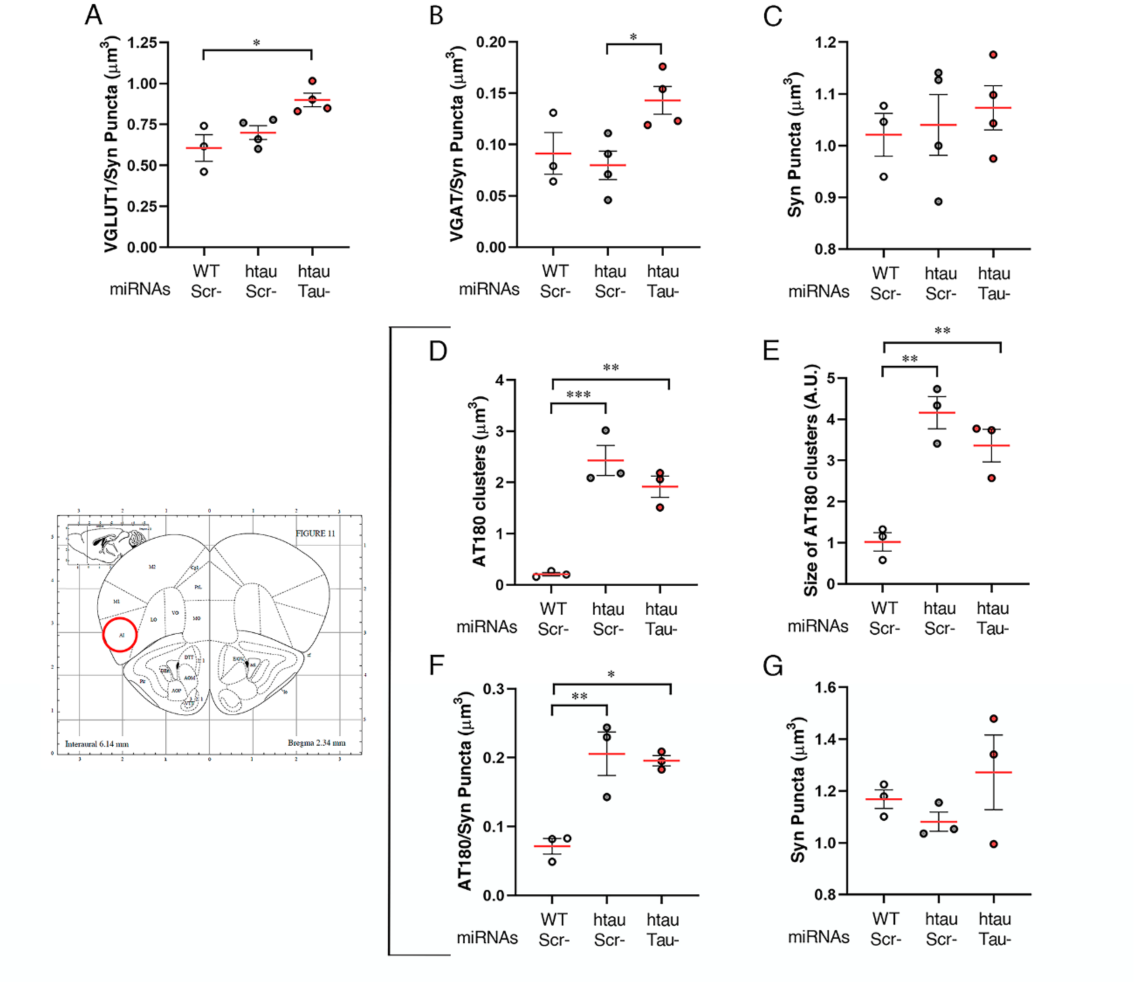

Figure S3. Tau reduction in the medial prefrontal cortex does not affect p-tau clusters in the agranular insular cortex, related to Figure 4.

A-C. Array tomography immunofluorescent labelling of glutamatergic (VGLUT1), GABAergic (VGAT) and synapsin 1a (Syn) puncta in the mPFC of miRNAs injected mice. Quantitative analysis of double-labeled puncta for Syn with (**A**) VGLUT1 or (**B**) VGAT and (**C**) total Syn+ puncta. WT Scr- n=3, htau Scr- n=4, htau Tau- n=4; *p<0,05, One-way ANOVA followed by Tukey's *post hoc* test. Data is shown as scatter dot plots, with mean ± SEM. **D-G.** Array tomography immunofluorescent labelling of p-tau (AT180) and synapsin 1a (Syn) puncta in the agranular insular cortex (AI) of miRNAs injected mice. Quantitative analyses of (**D**) density and (**E**) relative size of AT180 clusters in the AI. **F.** Density of AT180/synapsin colocalized puncta in the AI. **G.** Total Syn+ puncta. WT-Scr- n=3, htau-Scr- n=3, htau-Tau- n=3; *p<0,05, **p<0,01, ***p<0,001, One-way ANOVA followed by Tukey's *post hoc* test. Data is shown as scatter dot plots, with mean ± SEM.

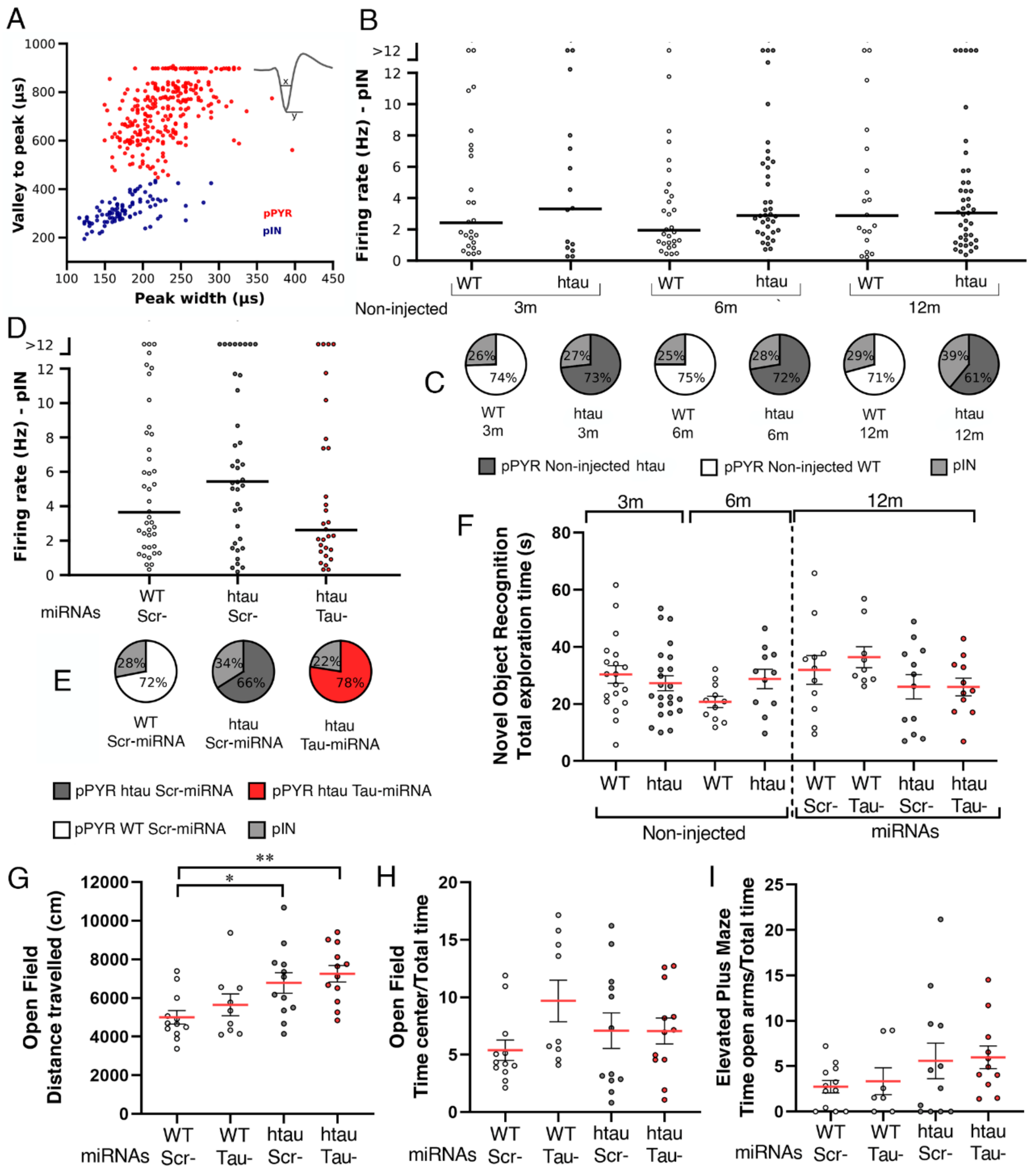

Figure S4. Tau-miRNA expression in the mPFC does not change firing rate of interneurons nor behavioural phenotypes in htau and WT mice, related to Figures 5 and 6*.*

A. Raster plot showing valley to peak and half amplitude duration of the mean spike waveform from each recorded neuron with a signal-to-noise ratio above 5. These features of spike waveforms sorted neurons in two clusters, corresponding to putative pyramidal neurons (pPYR, red dots) and putative interneurons (pIN, blue dots). **B.** Temporal course of firing rate of pIN in the mPFC of non-injected WT and htau mice at 3, 6 and 12 months of age. 3m: WT n=25 neurons/4 mice, htau n=15 neurons/3 mice, 6m: WT n=29 neurons/4 mice, htau n=36 neurons/4 mice, 12m: WT n=19 neurons/3 mice, htau n=41 neurons/6 mice; Mann-Whitney U test. Each dot represents the mean firing rate of each recorded neuron along the session. Black lines indicate the median value per group. **C.** Pie charts showing relative proportions of pPYR and pIN in the mPFC of non-injected mice. **D.** Firing rate of pIN in the mPFC for miRNAs injected mice at 12 months of age. WT Scr- n=41 neurons/4 mice, htau Scr-

n=38 neurons/5 mice, htau Tau- n=29 neurons/7 mice; Mann-Whitney U test. Each dot represents the mean firing rate of each recorded neuron along the session. Black lines indicate the median value per group. **E.** Pie charts showing relative proportions of pPYR and pIN in the mPFC of miRNAs injected mice. **F.** Total exploration time in the Novel Object Recognition test for non-injected (left) and miRNA injected mice (right). Non-injected: 3m: WT n=19, htau n=22, 6m: WT n=11, htau n=11, miRNAs injected: 12m: WT Scr- n=10, WT Tau- n=9, htau Scr- n=12, htau Tau-n=11; 3m and 6m: Unpaired t-test, 12m: One-way ANOVA. Data is shown as scatter dot plots, with mean ± SEM. **G-H.** Open field test**. G**) Total distance travelled and (**H**) time spent in the center relative to total time of exploration in the Open Field task for miRNAs injected mice. WT Scr- n=12, WT Tau- n=9, htau Scr- n=12, htau Tau- n=12; *p<0,05, **p<0,01, One-way ANOVA followed by Tukey's *post hoc* test. Data is shown as scatter dot plots, with mean ± SEM. **I.** Elevated plus maze test. Time spent in the open arms relative to total time of exploration by miRNAs injected mice. WT Scr- n=11, WT Tau- n=7, htau Scr- n=12, htau Tau- n=11; One-way ANOVA. Data is shown as scatter dot plots, with mean ± SEM.

Table S1. Mice used through the study, related to Methods.

Experimental group	htau Scr-miRNA												htau Tau-miRNA											
Mouse #	16	20	90	4	34	70	7	33	3	44	90	2	66	80	2	60	6	31	60'	11	76	74	49	66
NOR 3m	X	X	X	X	X	X	X	X	X	X	X	-	X	X	X	X	X	X	X	X	X	-	X	X
NOR 6m																								
NOR miR	X	X	X	X	X	X	X	X	X	X	X	X	X	X	X	X	X	X	X	X	X	X	X	-
PLUS MAZE miR	X	X	X	X	X	X	X	X	X	X	X	X	X	X	X	X	X	X	-	X	X	X	X	X
OPEN FIELD miR	X	X	X	X	X	X	X	X	X	X	X	X	X	X	X	X	X	X	X	X	X	X	X	X
PET miR							X	X	X	X	X	X					X	X	X	X	X	X	X	X
EF 3m																								
EF 6m																								
EF 12m																								
EF miR							X	X	X	X	X						X	X	X	-	X	X	X	X
AT miR							X	X		X	X						X	X			X	X		
AT miR AI							X	X			X						X	X			X			
WB 3m																								
WB 6m																								
WB miR		-	X	X	X	X			X				X	X	X	X			X	X			X	X
WB miR M1		X	X	X	X	X			X				X	X	X	X			X	X			X	X
SARKOSYL 3m																								
SARKOSYL 6m																								
SARKOSYL miR			X	X	X	X			X					X					X	X			-	X

Experimental group	WT Scr-miRNA												WT Tau-miRNA								
Mouse #	40	50	60	1	2	50'	2'	3	40'	10	20	30	1	2	3	40	55	60	1'	50	5
NOR 3m	X	X	X	X	X	X	X	X	X	X	X	X	X	-	X	X	-	X	X	X	X
NOR 6m																					
NOR miR	X	X	X	X	X	X	X	X	-	-	X	X	X	X	X	X	X	X	X	X	X
PLUS MAZE miR	X	X	X	X	X	X	X	X	-	X	X	X	X	X	X	-	X	X	X	X	-
OPEN FIELD miR	X	X	X	X	X	X	X	X	-	X	X	X	X	X	X	X	X	X	X	X	X
PET miR							X	X	X	X	X	X									
EF 3m																					
EF 6m																					
EF 12m																					
EF miR							X		X		X	X									
AT miR							X	X	X												
AT miR AI							X	X	X												
WB 3m																					
WB 6m																					
WB miR		X	X			X						X		X	X						
WB miR M1		X	X			X						X		X	X						
SARKOSYL 3m																					
SARKOSYL 6m																					
SARKOSYL miR				X	X																

Experimental group	WT 12m			htau 12m						WT 6m										
Mouse #	1	2	3	1	2	3	4	5	6	1	3	1´	5	*5´*	*3´*	*4´´*	*6*	*2´*	*6´*	*2´´*
NOR 3m																				
NOR 6m										X	X	X	X	X	X	X	X	X	X	X
NOR miR																				
PLUS MAZE miR																				
OPEN FIELD miR																				
PET miR																				
EF 3m																				
EF 6m										X	X	X	X							
EF 12m	X	X	X	X	X	X	X	X	X											
EF miR																				
AT miR																				
AT miR AI																				
WB 3m																				
WB 6m										X	X	X								
WB miR																				
WB miR M1																				
SARKOSYL 3m																				
SARKOSYL 6m										X	X	X								
SARKOSYL miR																				

Experimental group	htau 6m											WT 3m				htau 3m			KO
Mouse #	26	17	33	90	*89*	*5*	*97*	*37*	*46*	*31*	*84*	1	2	3	4	9	20	52	50
NOR 3m																			
NOR 6m	X	X	X	X	X	X	X	X	X	X	X								
NOR miR																			
PLUS MAZE miR																			
OPEN FIELD miR																			
PET miR																			
EF 3m												X	X	X	X	X	X	X	
EF 6m	X	X	X	X															
EF 12m																			
EF miR																			
AT miR																			
AT miR AI																			
WB 3m												X	X	X		X	X	X	X
WB 6m		X	X	X															X
WB miR																			X
WB miR M1																			X
SARKOSYL 3m												X	X	X		X	X	X	
SARKOSYL 6m		X	X	X															
SARKOSYL miR																			

Detail of the experiments performed with each mouse included in the study, as approved by the Institutional Animal Care and Use Committee of INGEBI-CONICET. Whenever possible the same breeds of mice were used for behavioral and post-mortem analyses. NOR: novel object recognition test; PET: positron emission tomography; EF: electrophysiological analysis; AT: array tomography immunofluorescence; WB: western blot of total tau; SARKOSYL: tau insolubility assay with sarkosyl reagent. mIR is for analyses made with miRNAs-injected mice and when not indicated is for non-injected groups (3, 6 and 12 months). Mouse # (mouse number) in *italics* were only used for behavioral experiments (not end point) and following the 3R rules were assigned to further experiments. AI: agranular insular cortex and M1: motor cortex 1. When not indicated, the biochemical analyses were performed in the PFC.

Table S2. Statistical analyses used through the study, related to Figures and Methods.

Figure	Graph	Data Structure	Type of test	*p*-values	Power (95% C.I. of diff)
Figure 1	1F	Normal Distribution	Unpaired t-test	0,0004 (***)	-1,155 to -0,491
Figure 1	1G	Normal Distribution	Unpaired t-test	0,0040 (**)	-1,180 to -0,305
Figure 1	1H	Non-normal Distribution	Two-sample Kolmogorov-Smirnov test	0,9999 (ns)	NA
Figure 1	1J, Anterograde	Non-normal Distribution	Mann-Whitney U test	0,6206 (ns)	-3,950 to 2,530
Figure 1	1J, Retrograde	Non-normal Distribution	Mann-Whitney U test	0,2004 (ns)	-4,850 to 1,030
Figure 1	1J, Stationary	Non-normal Distribution	Mann-Whitney U test	0,1763 (ns)	-1,240 to 7,410
Figure 1	1K	Normal Distribution	Unpaired t-test	<0,0001 (****)	0,029 to 0,061
Figure 2	2A	Normal Distribution	Two-way ANOVA; miRNAs factor	<0,0001 (****)	0,771 to 1,546
Figure 2	2C	Normal Distribution	Two-way ANOVA; miRNAs factor	<0,0001 (****)	-285,000 to -147,600
Figure 2	2D	Normal Distribution	Two-way ANOVA; miRNAs factor	<0,0001 (****)	24,490 to 68,880
Figure 2	2G	Non-normal Distribution	Mann-Whitney U test	<0,0001 (****)	-4,928 to -1,794
Figure 2	2H	Normal Distribution	Unpaired t-test	0,0170 (*)	0,464 to 4,634
Figure 3	3C, 3m	Normal Distribution	Unpaired t-test	0,0012 (**)	1,235 to 2,115
Figure 3	3C, 6m	Normal Distribution	Unpaired t-test	0,0125 (*)	0,535 to 2,461
Figure 3	3C, 12m	Normal Distribution	One-way ANOVA; Tukey's *post hoc* test	WT vs htau Scr-: <0,0001 (****) WT vs htau Tau-: 0,0004 (***) htau Scr- vs htau Tau-: 0,0261 (*)	WT vs htau Scr-: -2,583 to -1,221 WT vs htau Tau-: -1,786 to -0,571 htau Scr- vs htau Tau-: 0,082 to 1,365
Figure 3	3E, 3m	Normal Distribution	Unpaired t-test	0,0109 (*)	0,916 to 3,877
Figure 3	3E, 6m	Normal Distribution	Unpaired t-test	0,0050 (**)	1,300 to 3,850
Figure 3	3E, 12m	Normal Distribution	One-way ANOVA; Tukey's *post hoc* test	WT Scr- vs htau Scr-: 0,0250 (*) WT Scr- vs htau Tau-: 0,9587 (ns) htau Scr- vs htau Tau-: 0,0261 (*)	WT Scr- vs htau Scr-: -3,700 to -0,274 WT Scr- vs htau Tau-: -1,969 to 1,614 htau Scr- vs htau Tau-: 0,236 to 3,382
Figure 4	4B	Normal Distribution	One-way ANOVA; Tukey's *post hoc* test	WT Scr- vs htau Scr-: 0,0054 (**) WT Scr- vs htau Tau-: 0,9752 (ns) htau Scr- vs htau Tau-: 0,0026 (**)	WT Scr- vs htau Scr-: -0,396 to -0,086 WT Scr- vs htau Tau-: -0,143 to 0,166 htau Scr- vs htau Tau-: 0,109 to 0,396
Figure 4	4C	Normal Distribution	One-way ANOVA; Tukey's *post hoc* test	WT Scr- vs htau Scr-: 0,0219 (*) WT Scr- vs htau Tau-: 0,9505 (ns) htau Scr- vs htau Tau-: 0,0236 (*)	WT Scr- vs htau Scr-: -3,566 to -0,323 WT Scr- vs htau Tau-: -1,794 to 1,449 htau Scr- vs htau Tau-: 0,270 to 3,273
Figure 4	4D	Normal Distribution	One-way ANOVA; Tukey's *post hoc* test	WT Scr- vs htau Scr-: 0,0315 (*) WT Scr- vs htau Tau-: 0,8940 (ns) htau Scr- vs htau Tau-: 0,0445 (*)	WT Scr- vs htau Scr-: -0,222 to -0,012 WT Scr- vs htau Tau-: -0,122 to 0,089 htau Scr- vs htau Tau-: 0,003 to 0,198
Figure 5	5A, 3m	Non-normal Distribution	Mann-Whitney U test	0,7143 (ns)	-0,501 to 0,531
Figure 5	5A, 6m	Non-normal Distribution	Mann-Whitney U test	0,0600 (ns)	-0,008 to 0,758
Figure 5	5A, 12m	Non-normal Distribution	Mann-Whitney U test	0,0215 (*)	0,140 to 1,340

Figure 5	5B	Non-normal Distribution	Two-sample Kolmogorov-Smirnov test	For firing rates > 2 Hz 0,0115 (*)	NA
Figure 5	5D	Non-normal Distribution	Mann-Whitney U test	WT Scr- vs htau Scr-: 0,0087 (**) WT Scr- vs htau Tau-: 0,5888 (ns) htau Scr- vs htau Tau-: 0,0216 (*)	WT Scr- vs htau Scr-: 0,175 to 1,249 WT Scr- vs htau Tau-: -0,274 to 0,491 htau Scr- vs htau Tau-: -1,143 to -0,092
Figure 5	5E	Non-normal Distribution	Two-sample Kolmogorov-Smirnov test	For firing rates > 3 Hz WT Scr- vs htau Scr-: 0,0389 (*) WT Scr- vs htau Tau-: 1,0000 (ns) htau Scr- vs htau Tau-: 0,0365 (*)	NA
Figure 5	5F	Non-normal Distribution	Mann-Whitney U test	WT Scr- vs htau Scr-: 0,0121 (*) WT Scr- vs htau Tau-: 0,3242 (ns) htau Scr- vs htau Tau-: 0,0017 (**)	WT Scr- vs htau Scr-: 1,000 to 5,000 WT Scr- vs htau Tau-: -3,000 to 1,000 htau Scr- vs htau Tau-: -6,000 to -1,000
Figure 6	6B, 3m	Normal Distribution	Unpaired t-test	0,2753 (ns)	-12,480 to 3,652
Figure 6	6B, 6m	Normal Distribution	Unpaired t-test	0,0438 (*)	-21,540 to -0,334
Figure 6	6B, 12m	Normal Distribution	One-way ANOVA; Tukey's *post hoc* test	WT Scr- vs WT Tau-: 0,8821 (ns) WT Scr- vs htau Scr-: 0,0260 (*) WT Scr- vs htau Tau-: 0,8530 (ns) WT Tau- vs htau Scr-: 0,1692 (ns) WT Tau- vs htau Tau-: 0,4291 (ns) htau Scr- vs htau Tau-: 0,0022 (**)	WT Scr- vs WT Tau-: -12,560 to 22,040 WT Scr- vs htau Scr-: 1,639 to 33,890 WT Scr- vs htau Tau-: -21,370 to 11,540 WT Tau- vs htau Scr-: -3,581 to 29,630 WT Tau- vs htau Tau-: -26,580 to 7,275 htau Scr- vs htau Tau-: -38,390 to -6,956
Figure S1	S1A	Normal Distribution	One-way ANOVA; Dunnett's *post hoc* test	NI vs Scr-miRNA: 0,4595 (ns) NI vs 166-miRNA: 0,0200 (*) NI vs 724-miRNA: 0,0173 (*) NI vs 166+724-miRNAs: 0,0095 (**)	NI vs Scr-miRNA: -0,684 to 0,245 NI vs 166-miRNA: 0,103 to 1,031 NI vs 724-miRNA: 0,119 to 1,048 NI vs 166+724-miRNAs: 0,192 to 1,121
Figure S1	S1E	Normal Distribution	Unpaired t-test	0,0193 (*)	-27,710 to -2,670
Figure S1	S1F	Non-normal Distribution	Mann-Whitney U test	0,1610 (ns)	-967,500 to 147,300
Figure S1	S1G	Normal Distribution	Unpaired t-test	0,9678 (ns)	-4,986 to 5,188
Figure S2	S2C	Normal Distribution	One-way ANOVA; Tukey's *post hoc* test	WT vs htau Scr-: <0,0001 (****) WT vs htau Tau-: <0,0001 (****) htau Scr- vs htau Tau-: 0,2195 (ns)	WT vs htau Scr-: -3,747 to -1,867 WT vs htau Tau-: -3,090 to -1,331 htau Scr- vs htau Tau: -0,283 to 1,476
Figure S3	S3A	Normal Distribution	One-way ANOVA; Tukey's *post hoc* test	WT Scr- vs htau Scr-: 0,4758 (ns) WT Scr- vs htau Tau-: 0,0127 (*) htau Scr- vs htau Tau-: 0,0546 (ns)	WT Scr- vs htau Scr-: -0,314 to 0,126 WT Scr- vs htau Tau-: -0,513 to -0,073 htau Scr- vs htau Tau-: -0,403 to 0,004

Figure S3	S3B	Normal Distribution	One-way ANOVA; Tukey's *post hoc* test	WT Scr- vs htau Scr-: 0,8670 (ns)<br>WT Scr- vs htau Tau-: 0,1149 (ns)<br>htau Scr- vs htau Tau-: 0,0389 (*)	WT Scr- vs htau Scr-: -0,053 to 0,076<br>WT Scr- vs htau Tau-: -0,116 to 0,013<br>htau Scr- vs htau Tau-: -0,123 to -0,004
Figure S3	S3C	Normal Distribution	One-way ANOVA	0,7709 (ns)	NA
Figure S3	S3D	Normal Distribution	One-way ANOVA; Tukey's *post hoc* test	WT Scr- vs htau Scr-: 0,0007 (***)<br>WT Scr- vs htau Tau-: 0,0028 (**)<br>htau Scr- vs htau Tau-: 0,2725 (ns)	WT Scr- vs htau Scr-: -3,127 to -1,312<br>WT Scr- vs htau Tau-: -2,617 to -0,803<br>htau Scr- vs htau Tau-: -0,398 to 1,417
Figure S3	S3E	Normal Distribution	One-way ANOVA; Tukey's *post hoc* test	WT Scr- vs htau Scr-: 0,0017 (**)<br>WT Scr- vs htau Tau-: 0,0074 (**)<br>htau Scr- vs htau Tau-: 0,3039 (ns)	WT Scr- vs htau Scr-: -4,643 to -1,636<br>WT Scr- vs htau Tau-: -3,843 to -0,836<br>htau Scr- vs htau Tau-: -0,704 to 2,304
Figure S3	S3F	Normal Distribution	One-way ANOVA; Tukey's *post hoc* test	WT Scr- vs htau Scr-: 0,0072 (**)<br>WT Scr- vs htau Tau-: 0,0105 (*)<br>htau Scr- vs htau Tau-: 0,9331 (ns)	WT Scr- vs htau Scr-: -0,220 to -0,048<br>WT Scr- vs htau Tau-: -0,210 to -0,038<br>htau Scr- vs htau Tau-: -0,076 to 0,096
Figure S3	S3G	Normal Distribution	One-way ANOVA	0,3742 (ns)	NA
Figure S4	S4B, 3m	Non-normal Distribution	Mann-Whitney U test	0,8669 (ns)	-1,537 to 3,470
Figure S4	S4B, 6m	Non-normal Distribution	Mann-Whitney U test	0,0950 (ns)	-0,155 to 2,038
Figure S4	S4B, 12m	Non-normal Distribution	Mann-Whitney U test	0,8488 (ns)	-1,620 to 1,634
Figure S4	S4D	Non-normal Distribution	Mann-Whitney U test	WT Scr- vs htau Scr-: 0,2219 (ns)<br>WT Scr- vs htau Tau-: 0,3973 (ns)<br>htau Scr- vs htau Tau-: 0,0961 (ns)	WT Scr- vs htau Scr-: -0,686 to 3,718<br>WT Scr- vs htau Tau-: -2,217 to 0,971<br>htau Scr- vs htau Tau-: -4,326 to 0,265
Figure S4	S4F, 3m	Normal Distribution	Unpaired t-test	0,4519 (ns)	-11,240 to 5,098
Figure S4	S4F, 6m	Normal Distribution	Unpaired t-test	0,0535 (ns)	-0,135 to 16,240
Figure S4	S4F, 12m	Normal Distribution	One-way ANOVA	0,3325 (ns)	NA
Figure S4	S4G	Normal Distribution	One-way ANOVA; Tukey's *post hoc* test	WT Scr- vs WT Tau-: 0,7907 (ns)<br>WT Scr- vs htau Scr-: 0,0404 (*)<br>WT Scr- vs htau Tau-: 0,0059 (**)<br>WT Tau- vs htau Scr-: 0,3702 (ns)<br>WT Tau- vs htau Tau-: 0,1094 (ns)<br>htau Scr- vs htau Tau-: 0,8797 (ns)	WT Scr- vs WT Tau-: -2499 to 1214<br>WT Scr- vs htau Scr-: -3496 to -58,240<br>WT Scr- vs htau Tau-: -3972 to -534,400<br>WT Tau- vs htau Scr-: -2991 to 722,200<br>WT Tau- vs htau Tau-: -3467 to 246,100<br>htau Scr- vs htau Tau-: -2195 to 1243
Figure S4	S4H	Normal Distribution	One-way ANOVA	0,2077 (ns)	NA
Figure S4	S4I	Normal Distribution	One-way ANOVA	0,2886 (ns)	NA

Detail of data structure, statistical tests used, *p*-values and power per graph for all figures. Data structure was classified as Normal Distribution or Non-normal Distribution by Shapiro-Wilk test performed to each set of data. If the data passed the test (Normal Distribution), the statistical tests used for comparing groups were: Unpaired t-test (two groups, one independent variable), One-way ANOVA (three or more groups, one independent variable) or Two-way ANOVA (two independent variables). If the data did not pass the test (Non-normal Distribution), the statistical tests used for comparing groups were Mann-Whitney test and Two-sample Kolmogorov-Smirnov test. Approximate *p*-values are reported for each test and were classified according to the significant difference obtained between comparisons: (ns) no significant differences, (*) *p*-value<0,05, (**) *p*-value<0,01, (***) *p*-value<0,001 and (****) *p*-value<0,0001. The power, when applicable, indicates the 95% confidence interval of differences between the means (for Normal Distributions) and medians (for Non-normal Distributions). NA means no applicable.

Movies S1 and S2. Axonal transport in miRNAs treated human differentiated neurons, related to Figure 1.

Representative live-imaging movies used for analyzing axonal transport dynamics in Scr-miRNA (Movie S1) and Tau-miRNA (Movie S2) groups.
